# Proteomic Profiling Reveals a Specific Role for Translesion DNA Polymerase η in the Alternative Lengthening of Telomeres

**DOI:** 10.1016/j.celrep.2016.10.048

**Published:** 2016-11-08

**Authors:** Laura Garcia-Exposito, Elodie Bournique, Valérie Bergoglio, Arindam Bose, Jonathan Barroso-Gonzalez, Sufang Zhang, Justin L. Roncaioli, Marietta Lee, Callen T. Wallace, Simon C. Watkins, Patricia L. Opresko, Jean-Sébastien Hoffmann, Roderick J. O’Sullivan

**Affiliations:** 1 Department of Pharmacology and Chemical Biology, University of Pittsburgh Cancer Institute, School of Medicine, University of Pittsburgh, Pittsburgh, PA 15213, USA; 2 Department of Environmental and Occupational Health, University of Pittsburgh Cancer Institute, School of Medicine, University of Pittsburgh, Pittsburgh, PA 15213, USA; 3 Department of Cell Biology, University of Pittsburgh Cancer Institute, School of Medicine, University of Pittsburgh, Pittsburgh, PA 15213, USA; 4 CRCT, Université de Toulouse, Inserm, CNRS, UPS Equipe Labellisée Ligue Contre le Cancer, Laboratoire d’Excellence Toulouse Cancer, 2 Avenue Hubert Curien, 31037 Toulouse, France; 5 Department of Biochemistry and Molecular Biology, New York Medical College, Valhalla, NY 10595, USA

## Abstract

Cancer cells rely on the activation of telomerase or the alternative lengthening of telomeres (ALT) pathways for telomere maintenance and survival. ALT involves homologous recombination (HR)-dependent exchange and/or HR-associated synthesis of telomeric DNA. Utilizing proximity-dependent biotinylation (BioID), we sought to determine the proteome of telomeres in cancer cells that employ these distinct telomere elongation mechanisms. Our analysis reveals that multiple DNA repair networks converge at ALT telomeres. These include the specialized translesion DNA synthesis (TLS) proteins FANCJ-RAD18-PCNA and, most notably, DNA polymerase eta (Polη). We observe that the depletion of Polη leads to increased ALT activity and late DNA polymerase δ (Polδ)-dependent synthesis of telomeric DNA in mitosis. We propose that Polη fulfills an important role in managing replicative stress at ALT telomeres, maintaining telomere recombination at tolerable levels and stimulating DNA synthesis by Polδ.

## Graphical Abstract

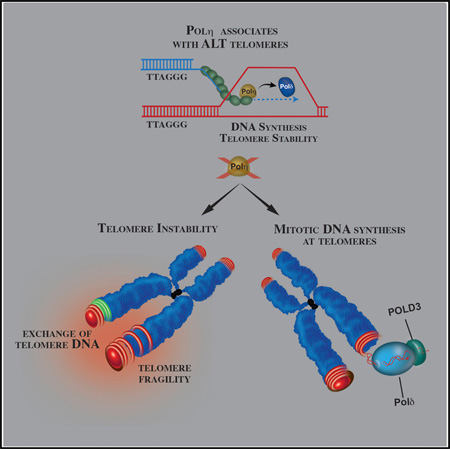

## INTRODUCTION

Telomere maintenance mechanisms are normally restrained to prevent tumorigenesis but are hijacked by cancer cells. Most cancer cells reactivate telomerase (Kim et al., 1994). However, telomerase is suppressed in a significant number of cancers that maintain telomere length by engaging the alternative lengthening of telomeres (ALT) mechanism (Bryan et al., 1997). ALT involves the inter-chromosomal exchange of telomeric DNA and is regulated by DNA repair and core recombination factors. These DNA repair factors frequently co-localize within clusters of telomeric DNA and specialized structures termed ALT-associated PML bodies (APBs) (reviewed in Cesare and Reddel, 2010). APB formation involves increased movement of telomeres within the nucleus to form clusters of 2–5 telomeres per APB (Draskovic et al., 2009). Elegant time-lapse imaging of telomeres in living cells has revealed that telomeric recombination and, by implication, so-called ALT activity can be stimulated by telomeric DNA damage, which triggers a homology search over distances of several microns in the cell and the clustering of several telomeres within PML bodies together with DNA repair and homologous recombination (HR) factors (Cho et al., 2014).

Following homology search, strand invasion, alignment, and synapsis, exactly how ALT cancer cells elongate their telomeres is unknown, but a model has been proposed that it proceeds by a specialized homology-directed repair (HDR) mechanism that might be similar to break-induced replication (BIR) (McEachern and Haber, 2006). How this process is initiated and coordinated is poorly defined, but telomeric DNA from a sister chromatid or DNA repair intermediate is envisioned as the template for copying and DNA synthesis during ALT (Cesare and Reddel, 2010). This model implies, and is supported by evidence, that ALT telomeres are prone to both spontaneous and chronic DNA damage that accumulates over successive cell cycles (Cesare et al., 2009; Lovejoy et al., 2012). The experimental induction of telomeric double-strand breaks (DSBs) (Cho et al., 2014) induces ALT activity that is likely primed from DNA repair intermediates. Similarly, ALT activity was transiently induced in ALT-negative HeLa LT (LT refers to long telomeres) and lung fibroblast cell lines through the co-depletion of the ASF1a and ASF1b (anti-silencing function 1a and 1b) histone chaperones, likely by deregulating chromatin assembly at stalled replication forks (O’Sullivan et al., 2014).

In normal and telomerase-positive cancer cells, DNA repair activities are prevented from associating with telomeres by the shelterin complex, which consists of TRF1, TRF2, RAP1, TIN2, TPP1, and POT1 (Palm and de Lange, 2008). To date, no deficiency in shelterin function has been linked with ALT activation or the elevated replicative stress and DNA damage observed at ALT telomeres (Lovejoy et al., 2012). Rather, the enhanced replicative stress at telomeres has been attributed to recurrent loss-of-function missense mutations in the histone H3.3-ATRX-DAXX chromatin assembly complex that have been cataloged in virtually every ALT-positive tumor (Heaphy et al., 2011; Schwartzentruber et al., 2012). Significantly, a causal role of ATRX mutations in ALT is supported by the observation that the reconstitution of wild-type ATRX in ALT cells suppresses ALT activity, likely by alleviating replicative stress at telomeres (Clynes et al., 2015). In addition, ALT telomeres contain an elevated frequency of variant C-type TCAGGG repeats (Conomos et al., 2012), which create high-affinity binding motifs for the NR2C1 and NR2F2 nuclear orphan receptor proteins within telomeres that then recruit the nucleosome remodeling deacetylase (NuRD) complex (Conomos et al., 2014; Déjardin and Kingston, 2009). By bridging outside of telomeres to their native binding sites within the chromosome body and promoting the insertion of telomeric DNA, these contribute to the genomic rearrangements and genomic instability that are common in ALT cancers (Marzec et al., 2015). Thus, ALT telomeres exist within an atypical chromatin and genomic configuration that sustains replicative stress, genomic instability, and ALT activity across successive generations of cancer cells.

In this study, we demonstrate the efficient capture, isolation, and proteomic profiling of human telomeres by proximity-dependent biotinylation (BioID) (Roux et al., 2012). In comparing the constituents of telomeres that employ distinct elongation pathways (telomerase versus ALT), we have identified that multiple DNA transactions, including DNA mismatch repair and translesion DNA synthesis proteins, converge uniquely at ALT telomeres. In pursuit of this mechanism, we uncovered a specific role for a specialized translesion DNA polymerase, DNA polymerase eta (Polη), in ALT regulation. Mutations in Polη have unequivocally been linked to xeroderma pigmentosum variant (XPV) syndrome an inherited disorder characterized by UV light sensitivity and skin cancer predisposition (Masutani et al., 1999). Though conventionally linked with translesion DNA synthesis of chromosomal and telomeric UV-induced cyclobutane pyrimidine dimers (CPD) lesions (Masutani et al., 1999; Pope-Varsalona et al., 2014), Polη also participates in the replication of structured DNA sequences such as common fragile sites (CFSs) (Bergoglio et al., 2013) and moonlights in recombination-coupled repair of collapsed replication forks (McIlwraith et al., 2005). Here, we provide evidence that this non-canonical role of Polη has been usurped to alleviate replicative stress and sustain recombination-associated DNA synthesis at ALT telomeres. We show that Polη is initially recruited to telomeres via RAD18-dependent ubiquitination of PCNA and partly via an association with the shelterin subunit TRF1. In the absence of Polη, we observe evidence of enhanced ALT activity that correlates with perturbed telomere replication. We also provide evidence of the coordinated action between Polη and Polδ regulates recombination-coupled DNA synthesis and thereby preserves telomere integrity.

## RESULTS

### Isolation and Profiling of Telomere Composition by BioID

**Figure 1:**
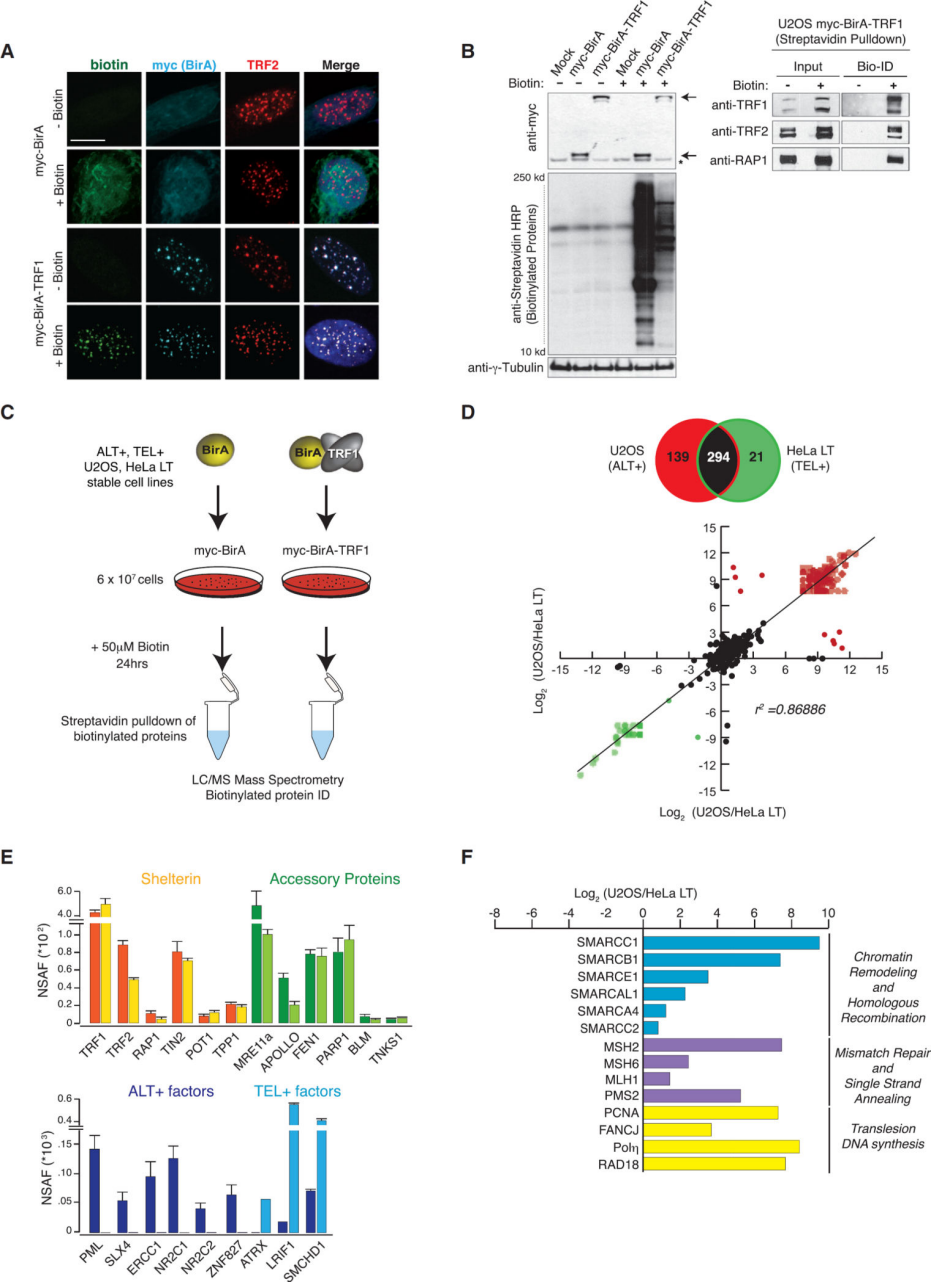
BioID Proteomic Profiling of Telomere Composition in ALT+ and TEL+ Cells (A) Immunofluorescence of myc-BirA and myc-BirA-TRF1 (cyan) localization in U2OS cells ± 50 µM biotin (green). TRF2 (red) staining provides a reference for telomere localization. DNA was counterstained with DAPI. Scale bar, 10 µm. (B) Protein biotinylation profile in mock transfected or myc-BirA and myc-BirA-TRF1 U2OS cells after biotin (50 µM, 24 hr) exposure and streptavidin pull-down. Arrows point to the myc-BirA fusion proteins. Asterisks (*) indicate a non-specific band in the myc blot. γ-Tubulin serves as a loading control. Blots on the right shows the shelterin proteins TRF1 (endogenous and myc-birA-TRF1), RAP1, and TRF2 as positive controls in pull-downs from myc-BirA-TRF1 U2OS-expressing cells. (C) BioID experimental approach for the identification by mass spectrometry of proteins in ALT+ (U2OS) and TEL+ (HeLa LT) cells. (D) Scatterplot of log_2_ (U2OS/HeLa LT) versus log_2_ (U2OS/HeLa LT) of the mass spectrometry results comparing TRF1-associated proteomics in ALT+ and TEL+ telomeres. (E) Normalized spectral abundance factor (NSAF) of proteins identified in ALT+ (dark-colored bars) and TEL+ (light-colored bars) telomeres. Examples include not only known shelterin and telomeric accessory proteins associated with both ALT+ and TEL+ telomeres but also specific ALT+ and TEL+ factors. (F) Examples of proteins with positive NSAF correlation (as log_2_ (U2OS/HeLa LT)) associated specifically with ALT+ telomeres and separated by their functional roles.

We employed BioID as a strategy to isolate and identify the telomeric proteome in ALT-positive (ALT+) U2OS cells and telomerase-positive (TEL+) HeLa cells with long telomeres (HeLa LT). BioID involves the expression of the promiscuous mutant prokaryotic biotin ligase BirA fused to a protein of interest (Roux et al., 2012). We fused BirA to TRF1, which binds exclusively and constitutively to TTAGGG repeats. We verified the co-localization between ectopically expressed myc-BirA-TRF1 and the endogenous shelterin protein TRF2 in both U2OS (Figure 1A) and HeLa LT cell lines (data not shown) while also observing the expected co-localization of myc-BirA-TRF1 and PML bodies, so-called ALT-associated PML bodies (hereafter referred to as APBs) only in ALT+ U2OS cells. In contrast, myc-BirA displayed a diffuse nuclear staining (Figures 1A and S1A). Furthermore, we verified that the stable expression of myc-BirA-TRF1 does not adversely affect the binding of endogenous TRF1 at telomeres and that the induction of BirA-mediated biotinylation does not perturb cell-cycle progression or provoke DNA damage checkpoint signaling in U2OS cells (Figure S1B–S1D).

In contrast to the diffuse pan-nuclear non-specific bio-tinylation pattern observed in myc-BirA-expressing cells, the addition of biotin (50 µM for 24 hr) stimulated BirA-mediated bio-tinylation that was largely restricted to telomeres, as determined by immunofluorescence in myc-BirA-TRF1-expressing U2OS cells (Figures 1A). This is consistent with the known 10-nm biotinylation radius of BirA (Kim et al., 2014). The selectivity of myc-BirA-TRF1 for telomeres was further shown by enrichment of shelterin proteins TRF1, TRF2, and RAP1 in direct streptavidin pull-down experiments and western blot with specific antibodies (Figure 1B). Therefore, the precise tethering of myc-BirA-TRF1 to telomeres ensures high-fidelity biotinylation of proteins localized directly at, or immediately adjacent to, telomeres (Figures 1A and S1A).

To isolate and compare the telomeric composition of ALT+ and TEL+ cancer cells, we performed streptavidin pull-downs from ~6 × 10^7^ myc-BirA-TRF1 and myc-BirA U2OS and HeLa LT cells that were equally supplemented with 50 µM biotin for 24 hr (Figure 1C). In total, we performed triplicate streptavidin pull-downs followed by liquid chromatography-mass spectrometry (LC-MS) for each sample and collated the listings of identified proteins in myc-BirA and myc-BirA-TRF1 immunoprecipitations (IPs). The proteins identified in myc-BirA IP-MS provided a negative reference sample in the mass spectrometry to identify and rule out non-specific associations (see Experimental Procedures for detailed description). In total, BioID identified 454 proteins, 294 of which were shared between ALT+ and TEL+ telomeres, with 139 and 21 proteins identified uniquely in ALT+ and TEL+, respectively (Figure 1D; Table S1). We compared the total protein cohorts retrieved by BioID with those retrieved by PICh (Proteomics of Isolated Chromatin segments) (Déjardin and Kingston, 2009) and QTIP (Quantitative Telomeric Chromatin Isolation Protocol) (Grolimund et al., 2013) methods to profile telomere constituents, and found a significant degree of overlap between BioID/PICh and BioID/QTIP (Figure S1E). The differences could be accounted for by the capacity of BioID to trace transient and temporal telomere proximal associations. With a Pearson correlation (*r*^2^) of ~0.87, protein identification by BioID proved to be highly specific and reproducible between technical replicates (Figure 1D). Therefore, BioID represents a relatively simple protocol to reproducibly isolate the telomeric proteome from its natural cellular context.

All six components of the shelterin complex (TRF1, TRF2, RAP1, TIN2, TPP1, and POT1) were retrieved at similar levels between ALT+ and TEL+ telomeres (Figure 1E). A greater proportion of TRF1 peptides were recovered due to self-biotinylation of the myc-BirA-TRF1 bait protein that is also captured in the streptavidin pull-down along with endogenous TRF1. In addition, several known shelterin accessory proteins (Palm and de Lange, 2008), including MRE11a, APOLLO, FEN1, PARP1, BLM, and Tankyrase 1, were recovered from ALT+ and TEL+ telomeres (Figure 1E). Importantly, we recovered proteins that are known to associate only with ALT+ (PML, SLX4, ERCC1, NR2C1, NR2C2, and ZNF827) and TEL+ (ATRX, LRIF1, and SMCHD1) telomeres, thereby demonstrating the capacity to capture distinct telomere states (Figure 1E). Notably, we did not retrieve peptides corresponding to several key telomere-associated proteins, including TERT, the catalytic subunit of telomerase, in TEL+ cells and RAD51, the central mediator of recombination, in ALT+ cells (Figure S1E). This could be accounted for by low relative abundance of these proteins and/or poor sequence coverage, paucity of available amines within target proteins, or loss during the extraction of biotinylated peptides.

The group of 139 proteins that uniquely associate with ALT+ telomeres contained a number with defined roles in transcription whose roles at telomeres are currently unknown (Figure 1F). Notably, we retrieved a significant number of proteins linked with distinct DNA repair activities, including several members of the SMARCAL (SWI/SNF related matrix-associated actin-dependent regulator of chromatin subfamily A-like) proteins that act in DNA strand annealing and HDR, including during ALT (Cox et al., 2016) (Figure 1F). Furthermore, we retrieved several proteins with well-characterized functions in DNA mismatch repair (MSH2, MSH6, MLH1, and PMS2).

Interestingly, we observed an enrichment of factors that functionally converge to regulate RAD18-mediated mono-ubiquitination of PCNA (^Ub-^PCNA) during translesion DNA synthesis: FANCJ, RAD18, and the specialized Y-family polymerase DNA Polη (reviewed in Sale et al., 2012) (Figure 1F). These data suggest that in addition to HR, other distinct DNA repair pathways converge at ALT+ telomeres. The questions of why and what their functions are remain unknown, though it appeared very likely that they could influence telomere function and their inhibition could have a significant impact on the ALT mechanism.

### Localization of DNA Polymerase η to ALT telomeres

**Figure 2:**
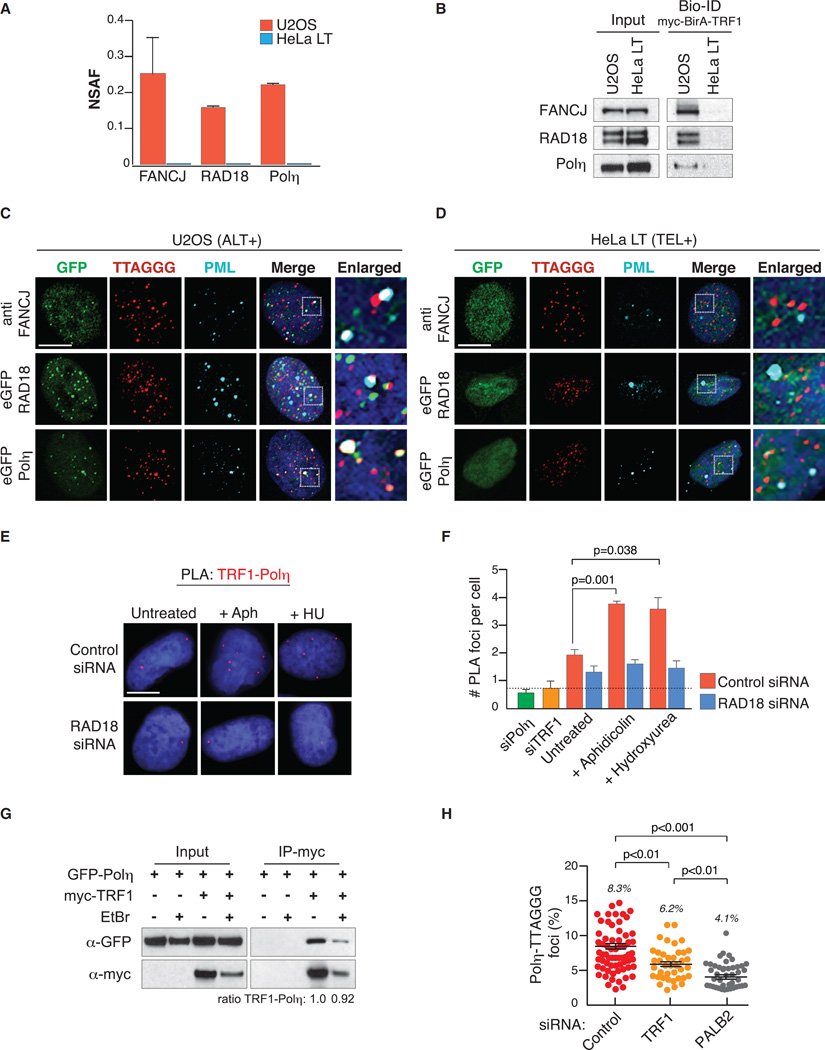
Multi-modal Recruitment of Polη to ALT+ Telomeres (A) NSAF values for FANCJ, RAD18, and Polη at ALT+ and TEL+ telomeres. (B) Western blot of FANCJ, RAD18, and Polη in TRF1 pull-downs from ALT+ and TEL+ telomeres. (C and D) Representative confocal images of the endogenous FANCJ or RAD18 and Polη GFP fusion proteins together with telomeres (TTAGGG) and PML in ALT+ and TEL+ cells. Co-distributions are shown in the enlarged areas. Scale bar, 10 µm. (E) Representative images of the proximity ligation assay (PLA) between TRF1-Polη in U2OS cells transfected with control or RAD18 siRNAs for 48 hr in the absence and presence of aphidicolin (Aph; 0.3 µM) or hydroxyurea (HU; 100 µM) for 24 hr. (F) Quantification of experiments performed in (E). Graph represents mean ± SD (n = 3, >600 cells per experiment) of the number of Polη-TRF1 foci per cell. (G) U2OS cells stably expressing myc alone or myc-TRF1 were infected with GFP-Polη adenovirus for 24 hr. Equal quantities of untreated and ethidium bromide (20 µg/mL) pre-treated cell lysates were used for immunoprecipitation (IP) with anti-myc beads. Western blots for Polη (GFP) and TRF1 (myc) are shown. (H) GFP-Polη distribution at telomeres in U2OS cells transfected with control, TRF1, or PALB2 siRNAs for 72hr. Data represent mean ±SEM (n ≥ 2) of the percent Polη-TTAGGG co-localization from the total telomere number. Significance was determined by Student’s t test and one-way ANOVA in (H).

To further examine the ALT specific association of TLS factors, we examined localization of FANCJ, RAD18, and Polη, since peptides of proteins were retrieved only in ALT+ cells (Figure 2A). We validated the mass spectrometry by directly probing elutes of U2OS and HeLa LT streptavidin pull-downs in western blots using antibodies against each protein (Figure 2B). We assessed their cellular localization in ALT+ (U2OS and SW26) and TEL+ (HeLa LT and SW39) cells (Figures 2C, 2D, and S2A) using antibodies against FANCJ (Déjardin and Kingston, 2009) and NLS-GFP-fusion proteins of RAD18 and Polη, which were used to determine subcellular localization of both proteins in response to UV-induced DNA damage and replicative stress (Bienko et al., 2010; Durando et al., 2013). In each case, we readily observed focal accumulation of FANCJ, GFP-RAD18, and GFP-Polη to telomeres in ALT+ U2OS and SW26 cells (Figures 2C and S2A), but not TEL+ HeLa LT and SW39 cells (Figures 2D and S2A). These co-localizations included but were not restricted to PML bodies. Significantly, expression of GFP tagged DNA polymerase κ (kappa), a related TLS polymerase, did not produce a similar telomeric localization as Polη (Figure S2A).

To probe for an association of the endogenous Polη with telomeres, we used proximity ligation assays (PLAs) with specific antibodies for Polη and TRF1 (Figure S2B). Indeed, telomere association of Polη was confirmed by PLA and was significantly enhanced following aphidicolin or hydroxyurea treatment of U2OS cells (Figures 2E and 2F). The RAD18-^Ub^PCNA-Polη interaction module has an established role in Polη recruitment during TLS and replication stress. The mono-ubiquitination of PCNA is mediated by the RAD18 ubiquitin E3 ligase (Bienko et al., 2005; Kannouche et al., 2004). In agreement with the involvement of RAD18-^Ub^PCNA in Polη localization, the TRF1-Polη PLA association was partially diminished by 30%–40% in U2OS cells that were depleted by small interfering RNA (siRNA) of RAD18. This effect was more pronounced in cells treated with aphidicolin or hydroxyurea, indicating that RAD18 is responsible for Polη recruitment to ALT telomeres upon replicative stress (Figures 2E, 2F, and S3A). Furthermore, the expression in U2OS cells of Polη mutants lacking the PCNA-interacting peptide (ΔPIP) or ubiquitin-binding domain (ΔUBZ), which are required for Polη focus formation (Bienko et al., 2005, 2010), completely abolished its telomeric localization (Figure S2C). The association of TRF1 with Polη, as determined by PLA, raised the possibility that Polη may interact directly with the shelterin complex. We infected U2OS cells stably expressing empty vector or myc-TRF1 with adenoviral GFP-tagged Polη, which allows for a low expression of Polη (Durando et al., 2013). Whole-cell lysates with or without ethidium bromide treatment to assay for DNA dependency were subjected to IP using myc-coupled beads. Blotting with GFP and myc antibodies revealed an association between and TRF1 (Figure 2G). Though ethidium bromide reduced the level of TRF1 in the IP, the association persisted indicating that the Polη-TRF1 association is not strictly mediated via DNA (Figure 2G).

Finally, several reports indicate that Polη can also be stabilized on chromatin by HR factors like RAD51 (McIlwraith et al., 2005) and PALB2 (Buisson et al., 2014), which are notably involved in the ALT mechanism. We expressed GFP-Polη in U2OS cells that were siRNA depleted of PALB2 and observed an ~50% decrease in Polη localization to telomeres (Figures 2H and S3A). In parallel, we performed the same analysis in cells depleted of shelterin component TRF1, which along with TRF2 orchestrates most processes at telomeres (Palm and de Lange, 2008). Depletion of TRF1 led to a small but significant reduction (~25%) in the telomeric localization of GFP-Polη (Figures 2H and S3A). In summary, these data illustrate that Polη is recruited to ALT telomeres in a manner that is largely mediated through the PIP and UBZ motifs within PCNA. However, downstream interactions with HR factors, including PALB2 and shelterin through TRF1, may stabilize Polη as it executes its activity in response to replicative stress at telomeres.

### The Impacts of Polη Depletion on ALT Activity

**Figure 3:**
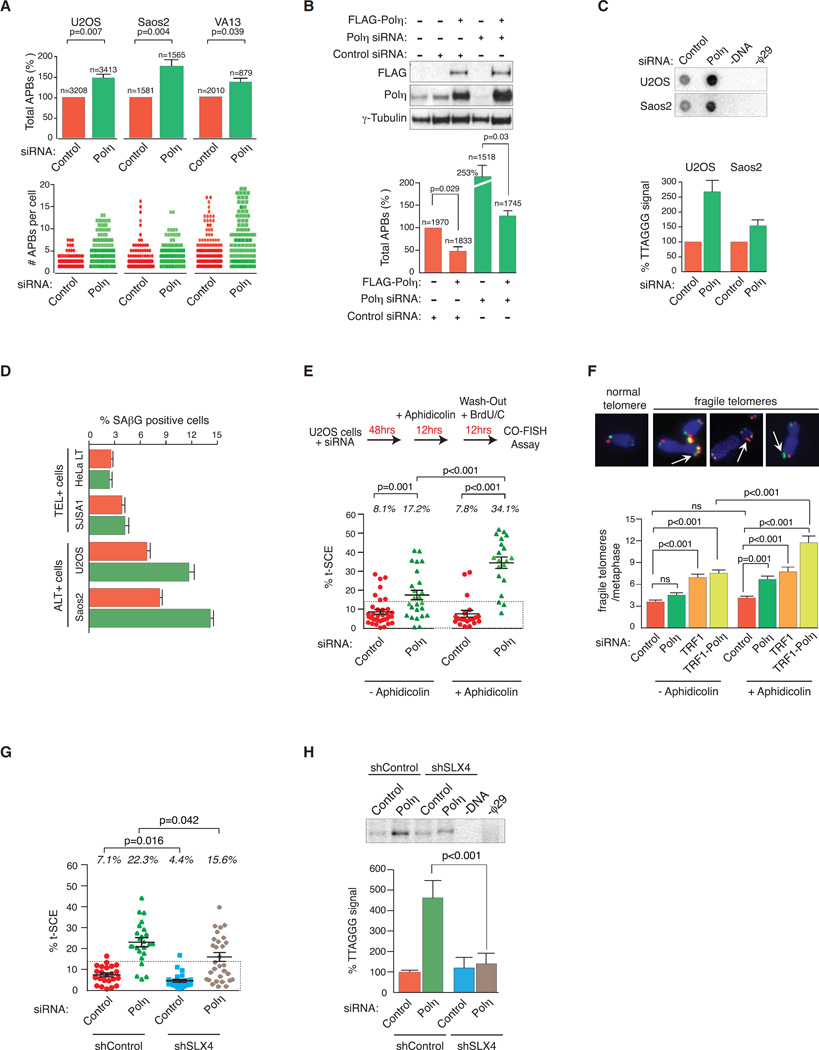
Enhanced ALT Activity and Replicative Defects following Polη Depletion in ALT Cells (A) APB analysis in U2OS, Saos2, and VA13 cells transfected with control and Polη siRNAs. APBs (telomere, PML, and RPA2 focico-localizations) were analyzed by IF-FISH. Data represent mean ± SEM of the normalized percentage of APB-positive cells. Data were also graphed beneath as number of APBs per cell. Statistical significance was determined using Student’s t test. (B) From top: western blots depicting ectopic expression of FLAG-Polη (WT) and knockdown of endogenous Polη with a single siRNA targeting the 3′ UTR of its mRNA. γ-Tubulin is a loading control. U2OS cells were treated as indicated, and APBs were scored as in (A). (C) C-circle assay in U2OS and Saos2 cells were transfected with control and Polη siRNAs. Quantification is relative to control siRNA samples ± SD (n = 3). (D) TEL+ cells; HeLa LT, SJSA1, and ALT+ cells; and U2OS, Saos2 cells were scored for percent senescence-associated β-galactosidase (SAβG) positivity 72 hr following transfection with control and Polη siRNAs. (E) Top: representation of the experimental procedure to induce replicative stress prior to CO-FISH assay. U2OS cells transfected with control and Polη siRNAs were cultured for 48 hr before aphidicolin (0.2 µM) was added for 12 hr and washed out prior to a final 12-hr pulse with 10 µM BrdU/BrdC. Bottom: data represent mean ± SEM (n = 3, >15 spreads per experiment) of the percentage of total t-SCEs in each metaphase. (F) Metaphases from control, Polη, TRF1, and Polη-TRF1 siRNA transfected U2OS cells were used to quantify telomere fragility. Examples of telomeres scores as control and fragile are shown. Data represent mean ± SEM of the number of fragile telomeres per metaphase. At least 25 metaphases were scored per condition. (G) U2OS shControl and U2OS shSLX4 cells were transfected with control and Polη siRNAs for 72 hr. T-SCEs were scored as in (D). (H) From the same cells used in (G) T-circle amplification protocol was conducted. Data represent mean ± SD of three independent experiments and is normalized to control shRNA and siRNA-treated cells. Significance was determined by Student’s t test.

To determine a potential function for Polη in the ALT mechanism, we first assessed the consequences of its absence by siRNA knockdown in U2OS, Saos2, and VA13 ALT+ cells. To begin, we quantified the number of APBs as defined by overlapping TTAGGG fluorescence in situ hybridization (FISH), PML, and RPA foci. In each cell line, at 72 hr after Polη depletion, we observed a significant increase of ~40% in the total percentage of APBs and the number of APBs per cell (Figure 3A). A similar increase was observed following depletion of RAD18 (Figure S3B). That this effect on ALT could be directly attributed to Polη depletion was demonstrated by (1) the depletion of Polη with a single siRNA targeting the 3′ UTR of its mRNA that also elicited a robust increase of total percentage of APB-positive cells in U2OS cells and (2) the suppression of this elevated APB phenotype when Polη expression levels were restored by ectopic expression of FLAG-tagged wild-type (WT) Polη cDNA in U2OS cells that were depleted of endogenous Polη by this single siRNA (Figure 3B). Interestingly, expression of FLAG WT-Polη in control siRNA-transfected cells led to an ~50% decrease of APBs (Figure 3B). These findings indicated that Polη might regulate other aspects of the ALT mechanism and prompted us to investigate other hallmarks of ALT activity.

For instance, the presence and accumulation of various species of extra-chromosomal telomeric repeat (ECTR) DNAs, like C-circles, provide a quantifiable readout of ALT activity (Henson et al., 2009). Indeed, Polη depletion provoked an elevation in C-circle levels in both U2OS and Saos2 cells but most significantly in the former, where an ~2- to 3-fold increase in C-circles was detected (Figure 3C). Previous studies have shown that the increased manifestation of these indicators of ALT activity negatively impacts cell viability, presumably by elevating genomic instability, and leads to the accumulation of senescence associated β-galactosidase-positive (SAβG+) cells within the cellular population (Conomos et al., 2014). Indeed, we found that knockdown of Polη led to increased SAβG+, although only in ALT+ (U2OS and Saos2) cells and not in TEL+ (HeLa LT and SJSA1) cells (Figure 3D). Telomere recombination in ALT generates telomere sister chromatid exchange (t-SCE) events that can be visualized using chromosome orientation FISH (CO-FISH). Following Polη depletion, t-SCEs increased from ~8% of total telomeres in control cells to ~17% in Polη-depleted U2OS cells (Figures 3E and S3D). This effect on t-SCEs was also clear in U2OS cells that we depleted of RAD18 (Figure S3C) and is similar to the observed effect of expressing an inactivated Polη on global sister chromatid exchange (Bergoglio et al., 2013).

Recent elegant work demonstrated that the expression, in ALT+ cells, of a TRF1 allele fused to the FokI nuclease (TRF1-FokI) can induce DSBs specifically within the telomeric duplex DNA (Cho et al., 2014). These DSBs are then repaired by HDR mechanisms and significantly enhance features of ALT, such as APB formation. Using this system, we induced wild-type and nuclease-dead TRF1-FokI-D450A in Polη siRNA-depleted U2OS cells. However, no additive APB formation was observed when we compared Polη and control siRNA-depleted U2OS cells (Figures S4A and S4B). This indicates that Polη does not act on telomeric DSBs that can induce ALT activity and is consistent with previous observations that concluded that Polη does not act on genomic DSBs induced by ionizing radiation (Kannouche et al., 2001).

Along with telomeric DSBs, replication stress has also been implicated in ALT activation. Polη was shown to alleviate replicative stress and facilitate the faithful replication through CFSs in response to exposure to the DNA polymerase inhibitor aphidicolin (Bergoglio et al., 2013). Therefore, we exposed ALT+ U2OS cells to a low dose (0.2 µM) of aphidicolin for 12 hr before preparing metaphase chromosomes for CO-FISH analysis of t-SCEs. The frequency of t-SCEs effectively doubled following aphidicolin treatment, with ~35% of total telomeres from Polη-depleted cells now displaying t-SCEs (Figure 3E). Thus, aphidicolin-induced replicative stress exacerbated telomere recombination that arises following Polη depletion, further substantiating the link between Polη and telomere replication in ALT cells. Telomeres also resemble CFSs in that the GC-rich tandem repeats can form structured DNA motifs like G-quadruplexes that impede fork progression and promote ATR-kinase-dependent replicative stress (Sfeir et al., 2009). TRF1 circumvents these problems and maintains telomere replication (Sfeir et al., 2009). Metaphase spreads were prepared from control and Polη-depleted cells that were exposed to low-dose (0.2 µM) aphidicolin for 12 hr and scored for fragile telomeres. The manifestation of fragile telomeres was significantly elevated following aphidicolin treatment of Polη-depleted cells (Figure 3F). This is reminiscent of the fragile telomere phenotype observed following TRF1 depletion (Sfeir et al., 2009) and co-depletion of TRF1 and Polη together with aphidicolin treatment significantly enhanced telomere fragility (Figure 3F). This suggests that Polη and TRF1 might cooperate to offset the deleterious consequences of replicative stress at ALT telomeres.

### SLX4 Promotes HDR at ALT Telomeres in the Absence of Polη

SLX4 (BTBD12 or FANCP) plays a critical role in DNA repair by acting as a scaffold for the structure-specific MUS81-EME1, XPF-ERCC1, SLX1, and GEN1 nucleolytic activities (Svendsen et al., 2009). SLX4 associated nucleases assemble can participate in the recovery of arrested and collapsed replicative forks late in S and G2/M phases of the cell cycle (Guervilly et al., 2015; Hanada et al., 2007; Wyatt et al., 2013). This can occur by cleavage of replication forks to generate double-strand breaks that act as substrates for homology-directed repair mechanisms (Hanada et al., 2007). Notably, SLX4 complex activities have also been implicated in telomere processing mechanisms, particularly at hyperextended telomeres like those of ALT+ cancer cells (Sarkar et al., 2015; Vannier et al., 2012; Wan et al., 2013). Therefore, we hypothesized that the higher frequency of t-SCEs in Polη-depleted cells could arise in an SLX4-complex-dependent manner.

To address this question, we generated stable SLX4 knockdown (SLX4 KD) cells by lentiviral small hairpin RNA (shRNA) transduction, achieving an 60% reduction of SLX4 mRNA (Figure S5A). Following 2 days of stable selection of transduced cells, we depleted Polη by siRNA in control shRNA and SLX4 depleted U2OS cells and assessed t-SCE frequency by CO-FISH. This confirmed our previous observation of elevated t-SCEs following Polη depletion. SLX4 KD alone partially reduced basal t-SCEs to 4.4%. Importantly, t-SCE frequency was reduced from 22.3% to 15.6% when Polη was depleted in SLX4 KD cells in which a greater number of metaphases harboring low t-SCE frequencies were readily apparent (Figure 3F). SLX4 has been shown to generate T-circles (TCs) that are suggested to be byproducts of D-loop or T-loop cleavage (Vannier et al., 2012; Wan et al., 2013). To assess whether Polη depletion leads to the SLX4-dependent generation of TCs, we performed T-circle amplifications (TCAs). Whereas knockdown of SLX4 had a marginal effect on TCs, Polη depletion yielded a strong increase in TC formation, which was suppressed by co-depletion of Polη and SLX4 (Figure 3G), indicating that in the absence of Polη, SLX4-mediated cleavage of replication intermediates promotes telomere sister chromatid exchange. We observed that the absence of this pathway further adversely impacts cellular viability, as co-depletion of both SLX4 and Polη elevated the percentage of SAbG+ U2OS cells from 8% to 12% (Figure S5B). These data are consistent with the evidence that the SLX4 complex is involved in the processing and recovery of unresolved replication intermediates as a means of genome preservation before cell division (reviewed in West et al., 2015).

### Polη Depletion Stimulates POLD3-Dependent Mitotic DNA Synthesis at Telomeres

Defective replication dynamics and the ensuing accumulation of under-replicated DNA within CFSs can provoke late DNA synthesis in G2 and M phases of the cell cycle (Bergoglio et al., 2013) that was recently determined as being dependent on the POLD3 subunit of Polδ (Minocherhomji et al., 2015). Our observations of increased fragile telomeres and telomere recombination suggest that Polη is required for DNA synthesis coupled restart of stalled forks within ALT cells. Furthermore, our previous studies have shown that the loss of Polη compromises chromosomal stability particularly during mitosis (Rey et al., 2009). Therefore, since late DNA synthesis of telomeres is a feature of ALT (Cho et al., 2014), perhaps due to BIR, we reasoned that Polη depletion could alter DNA synthesis programs at telomeres.

**Figure 4:**
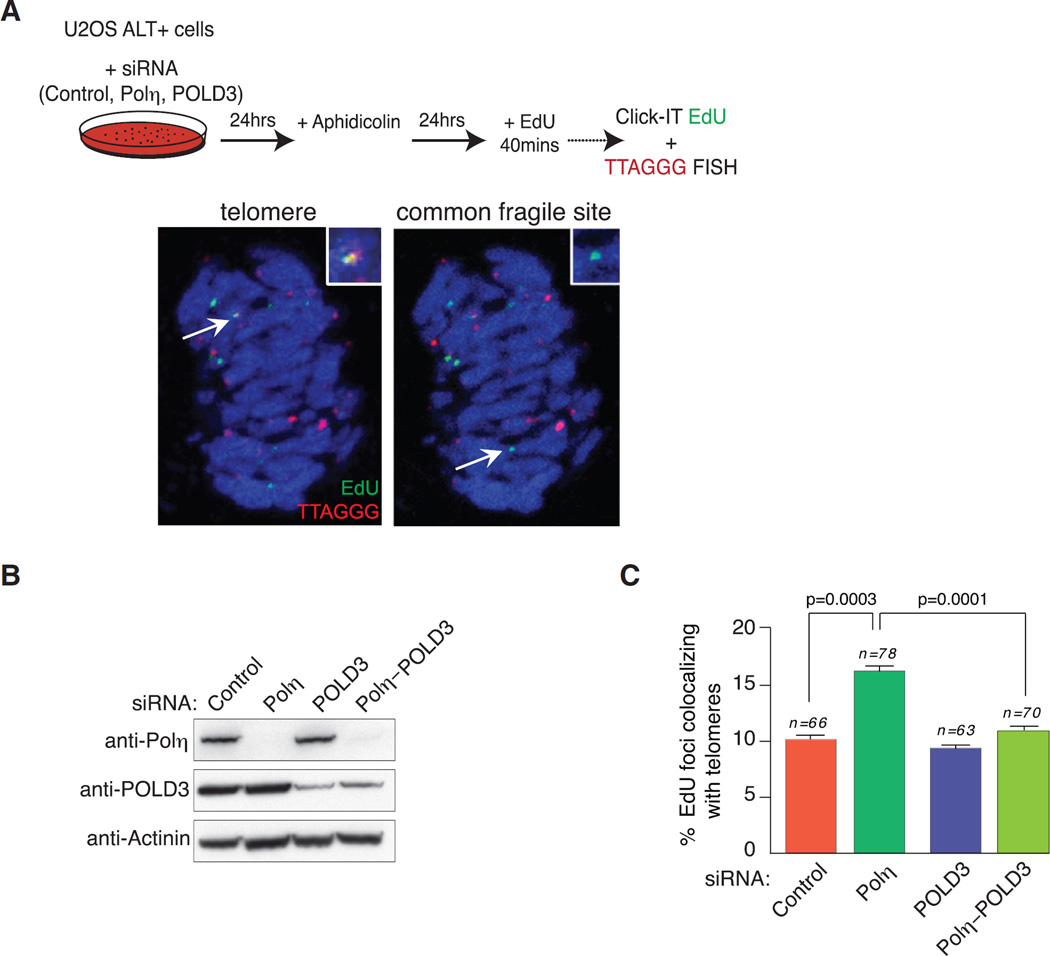
Polη Depletion Alters Replication Progression and Leads to Mitotic POLD3-Mediated DNA Synthesis at Telomeres (A) Experimental procedure to measure mitotic DNA synthesis at telomeres. U2OS cells transfected with control, Polη, POLD3, or both Polη and POLD3 siRNAs for 24 hr were treated for another 24 hr with aphidicolin and then for 40 min with EdU. EdU and TTAGGG FISH were detected on meta-phase spreads. Representative images of telomeres and common fragile sites are shown. (B) Western blots of Polη and POLD3 in U2OS cells mock-treated or transfected with Polη or POLD3 and both Polη-POLD3 siRNAs for ~48 hr. Actinin is used as a loading control. Protein extracts were generated 48 hr after transfection with siRNAs. (C) Quantifications of experiments performed in (A). Data represent mean ± SD of the percentage of EdU foci co-localizing with telomeres for the indicated number of metaphases analyzed. Significance was determined by Student’s t test.

For these experiments cells were treated with low dose of aphidicolin for 24 hr to induce replicative stress and telomere fragility, followed by a 40 min 5-ethynyl-2-deoxyuridine (EdU) pulse (Figure 4A). U2OS cells were transfected with siRNAs to deplete Polη and POLD3, individually and together (Figure 4B). Telomeres and EdU labeled newly synthesized DNA were then detected on metaphase chromosomes by a combination of TTAGGG FISH and EdU detection (Figure 4A). Overlapping TTAGGG and EdU signals on metaphase chromosomes were then enumerated. Importantly, a majority of chromosomal EdU foci reflecting common fragile site expression following aphidicolin treatment as would be expected (Figure 4A). These experiments revealed a significant increase of EdU foci co-localizing with telomeres in Polη but not POLD3 depleted cells (Figure 4C). Strikingly, the co-depletion of Polη and POLD3 completely reversed the effects of Polη depletion alone (Figure 4C). Therefore, replicative stress stimulates mitotic DNA synthesis at ALT telomeres that we now show to be suppressed by Polη.

### Polη Can Initiate DNA Synthesis as a Precursor to Polδ-Mediated Extension of Telomeric DNA

It has been established that Polη moonlights in non-TLS related pathways, notably homologous recombination (HR) mechanisms like strand displacement (McIlwraith et al., 2005). It was shown that recombinant Polη preferentially extends the invading strand of an artificial oligo-based D-loop, compared to replicative DNA polymerases and TLS Polι (McIlwraith et al., 2005). Nuclear extracts from XPV cells are severely deficient in this D-loop extension activity (McIlwraith et al., 2005). Subsequent studies indicated that recombinant Polη can extend the invading strand of plasmid-based D-loops, but this activity was PCNA independent, whereas Polδ extension required PCNA (Sebesta et al., 2013). Furthermore, the DNA synthesis activity of Polη at D-loops is stimulated by its interaction with RAD51 and PALB2 (Buisson et al., 2014; McIlwraith et al., 2005), which we found enhance the localization of Polη to telomeres (Figure 2H). These data suggested that Polη functions in HR and ALT by regulating DNA synthesis from D-loops that arise due to deregulated replication dynamics at ALT telomeres.

**Figure 5:**
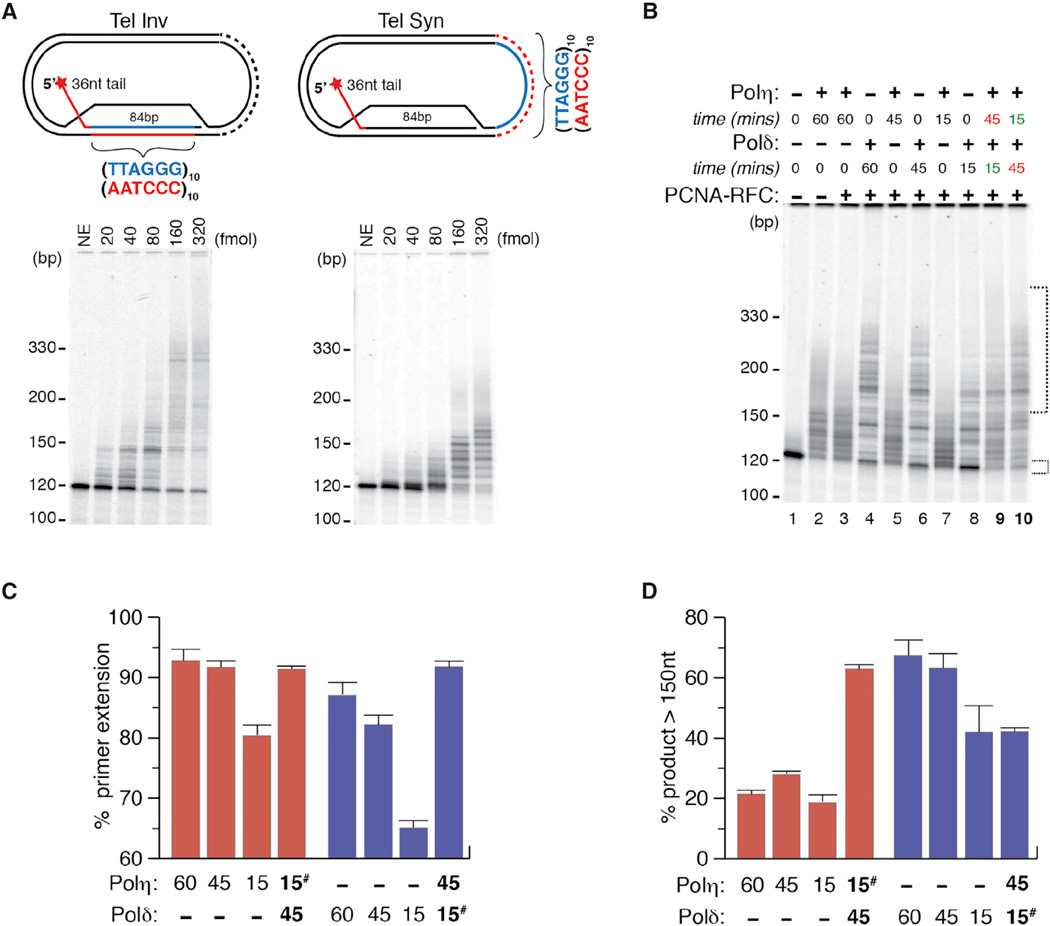
Initiation of Strand-Displacement-Coupled DNA Synthesis by Polη (A) Plasmid-loop systems used in this study. Tel invasion (Tel Inv) D-loop system is shown on the left, while Tel synthesis (Tel Syn) is shown on the right. Below each D-loop system is a representative image of Polη-mediated extension reactions at increasing enzyme concentrations. DNA molecular weight is shown on the left of each gel to show the length of the extension. (B) Representative image of extension products from Tel-Syn D-loop system in reactions containing purified Polη and/or Polδ for different times and in the presence or not of the accessory complex PCNA-RFC. (C) Quantifications of the percentage of primer used i.e. % of unused primer was used to estimate the total strand displacement (lower small bracket) and (D) the percentage of products > 150nt (large broken brackets) from the assays performed in (B). Lanes where both Polη and Polδ were added to the reactions are highlighted in bold and # refers to the polymerase which was added first.

To investigate this, we prepared plasmid-based mobile D-loops with ten tandem TTAGGG repeats either in the invading strand (Tel-Inv), so that the displaced region is telomeric, or downstream of the invading strand, so that the (3′-AATCCC-5′) repeats serve as templates for subsequent DNA synthesis (Tel-Syn) (Figure 5A). First, the addition of increasing amounts (20–320 fmol) of recombinant protein revealed that Polη very efficiently initiates and conducts strand displacement DNA synthesis on the Tel Inv D-loop, in agreement with previous reports (McIlwraith et al., 2005). However, the insertion of telomeric sequences downstream of the D-loop in Tel-Syn significantly impeded progression of Polη-mediated DNA synthesis (Figure 5A). This suggested that Polη might only be required to initiate DNA synthesis and to generate short products before handing off to Polδ.

To further probe a potential coordination between Polη and Polδ on telomeric DNA, we performed a series of assays using the Tel-Syn substrate given its greater in vivo relevance. We measured the percentage of unused primer in the D-loop and the percentage of products extended by more than 30 nt (total length with primer, ~150 nt and longer). These measurements provide comparable quantitative readouts of the strand displacement activity and progression of DNA synthesis, respectively. Though the strand displacement activity of Polη does not strictly require PCNA, Polδ depends on RFC-PCNA for its assembly on D-loop substrates (Sebesta et al., 2013). The greater strand displacement activity of Polη was readily apparent, even though Polδ displayed greater DNA synthesis progression and generated longer telomeric extension products than Polη (Figure 5B, lanes 2 and 4; Figures 5C and 5D). This is most clearly observed in 15-min reactions, where Polη displaced ~80% of the D-loops although only ~20% of the products were extended greater than 30 nt (red bars). In contrast, 50% of the products generated by Polδ were greater than 30 nt despite ~65% D-loops having been extended (blue bars) (Figure 5B, lanes 7 and 8; Figures 5C and 5D). Finally, we conducted 60-min reactions in which the order of addition and duration of polymerase activity were alternated (Figure 5B, lanes 9 and 10). With respect to final strand displacement activity, no noticeable difference was observed when either polymerase was added first (Figure 5C). In contrast, the order of polymerase incubation had a significant effect on the progression of DNA synthesis from Tel-Syn. We found that the initial incubation of Tel-Syn with Polη for 15 min before adding Polδ for a further 45 (Figure 5B, lane 10) generated ~25% more products extended greater than 30 nt compared to the inverse, when Polδ was added before Polη (Figure 5B, lane 9; Figure 5D). Our biochemical studies, using substrates that mimic replication fork repair intermediates, support the proposed model (Buisson et al., 2014), where during the restart of stalled replication forks, Polη initiates recombination associated DNA synthesis at D-loops before the substitution of Polδ enhances DNA synthesis to generate longer tracts of telomeric DNA.

## DISCUSSION

In this study, we applied BioID-based proteomics to isolate and profile telomere composition in cancer cells that utilize distinct telomere maintenance mechanisms: telomerase or the ALT mechanism. Our comparative analysis revealed that proteins from distinct DNA repair pathways converge at telomeres that engage the ALT mechanism. These include not only factors involved in DNA replication, homologous recombination, and mismatch repair but also proteins conventionally linked with translesion DNA synthesis (TLS), like Polη, which has not been previously linked with the ALT mechanism.

In addition to its canonical TLS role in the bypass of UV-light-induced DNA lesions, Polη has been shown to have non-canonical functions to repair damage arising from cell-intrinsic sources. These could provide a rationale for its association with ALT telomeres and potential regulatory role within the ALT mechanism. These include the replication of non-B-form, GC-rich, structured DNAs found at CFSs (Bergoglio et al., 2013; Rey et al., 2009). The depletion of Polη results in the accumulation of under-replicated DNA and subsequent defects in mitosis due to CFS instability (Bergoglio et al., 2013; Rey et al., 2009). The faithful replication of common fragile sites is thought to involve HR-associated mechanisms that restart stalled replication forks. This link with HR is supported by evidence of Polη focus formation and interactions with essential HR factors, like RAD51 and PALB2, upon replicative stress (Buisson et al., 2014; Durando et al., 2013; McIlwraith et al., 2005). Furthermore, in vitro studies have established that recombinant Polη preferentially initiates DNA synthesis from synthetic D-loop substrates, providing a structural context in which Polη might function during HR in vivo (Buisson et al., 2014; McIlwraith et al., 2005; Sebesta et al., 2013).

Considering these findings with the current models of the ALT mechanism, in which HR is strongly implicated, the non-canonical function of Polη in HR would appear most pertinent with respect to Polη’s role at ALT telomeres. Consistent with this, we found that the depletion of Polη leads to elevated evidence of ALT activity, as demonstrated by an elevated presence of not only ALT-specific markers like APBs and ECTR but also more frequent fragile telomeres and t-SCEs, which reflect compromised replication and enhanced recombination. This increased frequency of t-SCEs could be partially suppressed when SLX4 was co-depleted together with Polη. The SLX4 nucleolytic complex has been shown to cleave unresolved stalled forks that persist and collapse in late S and G2/M phases of the cell cycle (Hanada et al., 2007; Wyatt et al., 2013). This could generate a one-ended DSB that can prime repair of the fork, potentially by break-induced replication (BIR), a unique form of HR that has implicated as a principle driver of telomere elongation in ALT cells (McEachern and Haber, 2006; West et al., 2015). One aspect that makes such HR-mediated repair by BIR distinct from more conventional HR mechanisms is that DNA synthesis during BIR is not restricted to S phase (McEachern and Haber, 2006) and depends on the POLD3 subunit of the replicative polymerase, Polδ (Costantino et al., 2014; Minocherhomji et al., 2015). In agreement with the involvement of POLD3 in this process, we found that the depletion of Polη leads to late DNA synthesis at telomeres during mitosis, which is also dependent on POLD3. Taken together, we propose that Polη functions within a HR mechanism involved in the restart of stalled replication forks, and this is required to preserve the structural integrity of telomeres and avoid the potentially detrimental mutagenic effects of unrestrained DNA synthesis that might occur via BIR (see below).

However, there are alternative scenarios that could explain the enhanced ALT activity and defects in telomeres that manifest upon Polη depletion. First, Polη has been implicated in the replication through G-quadruplex (G4) structures (Bétous et al., 2009). These structures exhibit a propensity to form within GC-rich DNA like that found within the telomeric TTAGGG sequence and can be induced by aphidicolin treatment, leading to replicative stress and structural abnormalities within telomeres (Biffi et al., 2013). However, the presence of multiple helicases that can unwind G4s at ALT telomeres, like BLM and FANCJ that were identified in BioID, would argue that there are redundant modes to prevent the accumulation of G4s that might arise upon Polη depletion. Second, together with PCNA and the MSH2-MSH6 mismatch repair complex, Polη has been implicated in the repair of clustered oxidized DNA lesions (Zlatanou et al., 2011), and in vitro studies have shown that recombinant Polη can bypass 8-oxo-guanine with a high degree of accuracy (McCulloch et al., 2009). ALT+ tumors harbor elevated levels of reactive oxygen species owing to mitochondrial dysfunction (Hu et al., 2012), and unrepaired oxidative lesions could adversely affect the integrity of telomeres or shelterin binding (Opresko et al., 2005). As with Polη, we found by BioID that MSH2-MSH6 specifically associates with ALT telomeres. The molecular function of these proteins within the ALT mechanism remains unknown. Even though Polη-deficient cells derived from XPV patients are proficient in base excision repair (BER) and nucleotide excision repair (NER), it would be of interest to identify and measure oxidative damage at ALT telomeres and explore in future studies whether Polη-MSH2-MSH6 are involved in regulating this.

### The Pros and Cons of Polη Use at ALT Telomeres

There is relatively little known in relation to the polymerases that regulate the ALT mechanism despite an appreciation that it involves mechanisms that are distinct from normal DNA replication (McEachern and Haber, 2006). Building on previous work (Buisson et al., 2014; McIlwraith et al., 2005; Sebesta et al., 2013), we show here that Polη can more efficiently initiate DNA synthesis from telomeric D-loops than Polδ. However, we found that further Polη-mediated DNA synthesis is stifled on telomeric DNA templates and therefore might involve a switch to Polδ. This notion is consistent with evidence indicating the involvement of multiple polymerases during HR (Moldovan et al., 2010; Sebesta et al., 2013). This raises the question as to how such a polymerase switch might occur. Mechanisms of polymerase switching in HR are currently unknown but may be related to those involved in TLS polymerase switching past bulky DNA adducts, where again multiple polymerases are involved (McCulloch et al., 2004). Here, RAD18-dependent ubiquitination of PCNA induces the recruitment of Polη to bypass the lesion. The recruitment of Polη could also be facilitated through an interaction with the POLD2 subunit of Polδ (Baldeck et al., 2015). The TLS polymerases are removed via de-ubiquitination of PCNA and replaced by Polδ. Deciphering whether the polymerases directly interact or are bridged through their associations with PCNA will be a key issue to resolve in future studies.

Another key question relates to understanding the possible benefits for utilizing Polη in addition to replicative polymerase Polδ. The hyperextension of telomeres in ALT cells may have driven an adaptation that co-opted additional polymerases to manage recombination coupled repair of stalled replication forks at ALT telomeres. In addition, while PCNA recruits Polη to telomeres in vivo, recombinant Polη, unlike recombinant Polδ, does not require additional factors to initiate DNA synthesis from synthetic telomeric D-loop templates (Sebesta et al., 2013), providing another potentially important advantage for sequestering Polη to facilitate the replication of hyperextended ALT telomeres.

Finally, even though Polη seems to be involved in preserving the integrity of the ALT mechanism, Polη primed DNA synthesis could conceivably come with the additional cost of introducing mutations in ALT telomeric DNA. Polη lacks an intrinsic proofreading activity and can insert discrepant nucleotides in undamaged DNA at remarkably high frequency compared to other poly-merases (Arana and Kunkel, 2010; Matsuda et al., 2000). This can result in T-C base substitutions at a rate of 1 per 18 nucleotides (Matsuda et al., 2000). Should such T-C transitions in the TTAGGG repeat occur, it could generate TCAGGG repeats that are known to be enriched at ALT telomeres and recruit orphan nuclear receptor proteins that drive intra-chromosomal contacts and genome rearrangements (Marzec et al., 2015). The switch from Polη to Polδ might provide some extrinsic proofreading mechanism where Polδ could minimize errors made by Polη, as has been shown in the context of TLS of bulky DNA lesions (Nick McElhinny et al., 2006). Nonetheless, utilizing Polη over short stretches of telomeric DNA as a means to efficiently restart stalled replication forks would be more favorable than the hyper-mutagenesis and genomic rearrangements associated with POLD3-dependent mitotic DNA synthesis like in BIR (Costantino et al., 2014). Future studies will continue to examine the potential interactions and roles that both canonical and TLS polymerases could have in regulating the ALT mechanism.

## EXPERIMENTAL PROCEDURES

Detailed experimental procedures are provided in Supplemental Experimental Procedures.

### BioID of Telomere-Associated and Proximal Proteins

U2OS and HeLa cells stably expressing myc-BirA alone or myc-BirA-TRF1 were generated by retroviral infection with particles generated from amphotropic 293T viral packaging cell lines. Infected cells were selected using puromycin (2 µg/mL) for 5–7 days, and stable protein expression was validated by western blot and immunofluorescence. Proximity-dependent biotinylation and streptavidin capture of biotinylated proteins was performed as described in (Roux et al., 2012) with minor modifications (see Supplemental Experimental Procedures).

### Telomere Detection Assays

Immunofluorescence (IF)-FISH for APBs, C-/T-circle assays, and CO-FISH as performed previously (O’Sullivan et al., 2014) were used to study ALT activity and T-SCE.

### Statistical Methods

Statistical analysis was performed using Excel and GraphPad Prism. Significance was determined by applying the Student’s t test throughout and one-way ANOVA for Figure 2H.

## Supplementary Material

2

## Highlights

- Utilize BioID to profile the composition of telomeres in cancer cells
- DNA polymerase eta (Polη) localizes specifically to ALT telomeres
- Loss of Polη enhances ALT and promotes mitotic DNA synthesis at telomeres

## In Brief

Garcia-Exposito et al. use BioID to determine the composition of telomeres in human cancer cells that rely on distinct telomere elongation mechanisms to survive. They suggest that the translesion polymerase DNA polymerase eta (Polη) regulates the recombination-associated alternative lengthening of telomeres (ALT) mechanism.
