# Supplementary material for: Proteomic Profiling Reveals a Specific Role for Translesion DNA Polymerase η in the Alternative Lengthening of Telomeres
Source: Cell Rep. Author manuscript; Available in PMC 2017 Apr 26. (PMC5406014; doi:10.1016/j.celrep.2016.10.048)
Supplement: 2 [file NIHMS827343-supplement-2.pdf]

Figure S1.

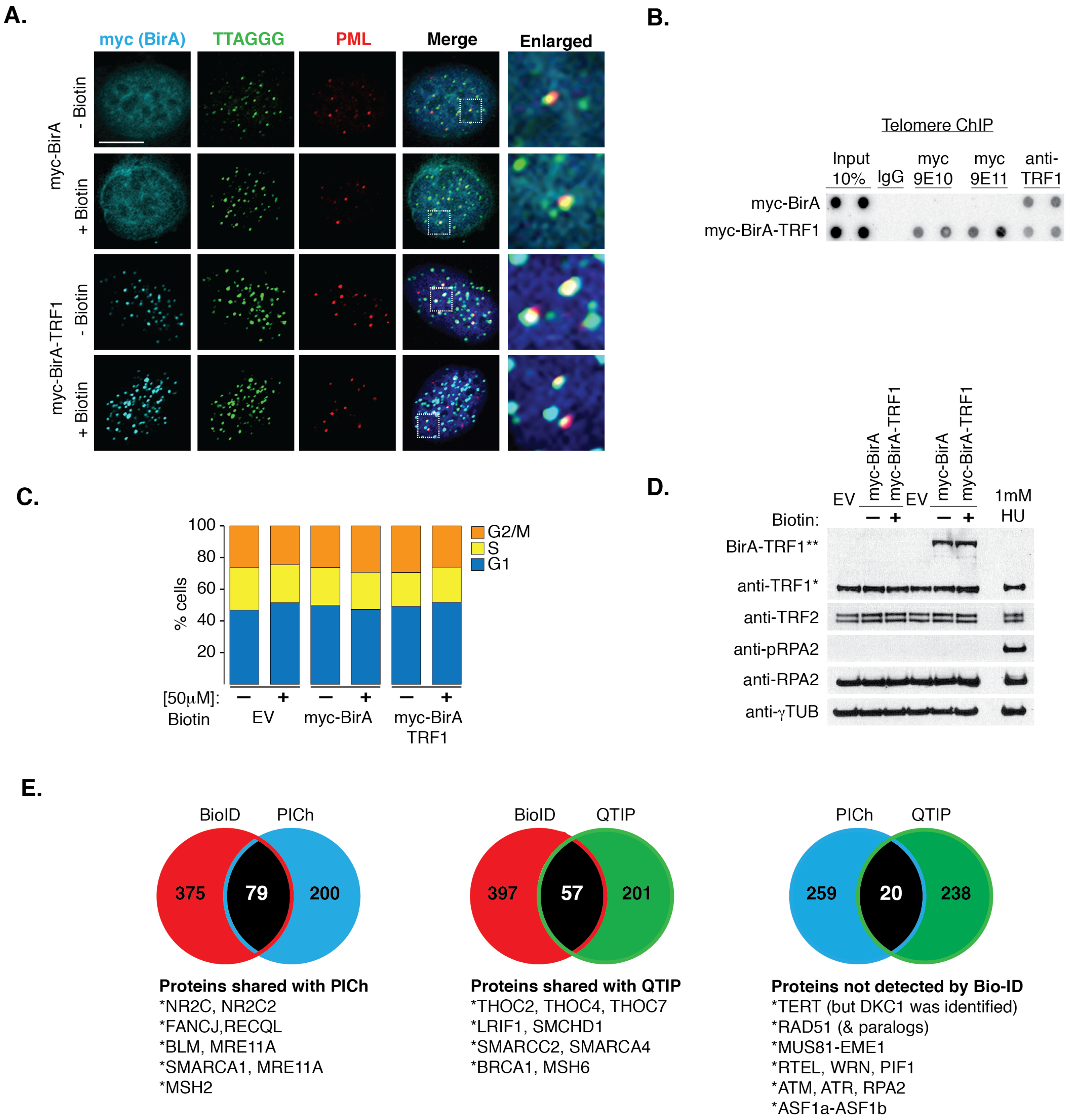

Figure S1. Related to Figure 1. BirA-TRF1-mediated protein biotinylation at telomeres. (A) Representative confocal images of U2OS-myc-BirA or U2OS-myc-BirA-TRF1 stable cells together with telomeres (TTAGGG) and PML in the presence or absence of biotin (50µM, 24hrs). Enlarged areas showing myc-TTAGGG-PML co-distributions are shown. Scale bar,10µM. **(B)** ChIP for telomeric DNA associated with endogenous TRF1 and myc-TRF1 fusion protein in U2OS cells stable for myc-BirA or myc-BirA-TRF1. Total TRF1 is shown in inputs. **(C)** Cell cycle analysis in U2OS control or stable for myc-BirA or myc-BirA-TRF1 in the presence or absence of Biotin. Bars represent the percentage of cells in G1, S or G2/M in each experimental condition. **(D)** Western blot analysis of DNA damage response in U2OS cells transfected with an empty vector (EV) or stable for myc-BirA or myc-BirA-TRF1 as shown in **(C)**. * Indicates endogenous TRF1 and ** indicates myc-BirA-TRF1 fusion protein. U2OS cells treated with 1mM hydroxyurea for 4hrs is used as positive control for DNA damage induction. γ-Tubulin is used as a loading control. **(E)** Overlapping comparisons of proteins retrieved by BioID, PICH and QTIP proteomic approaches. Examples of proteins shared between BioID/PICh and BioID/QTIP and proteins detected by PICh or QTIP but not by BioID are shown below charts.

Figure S2.

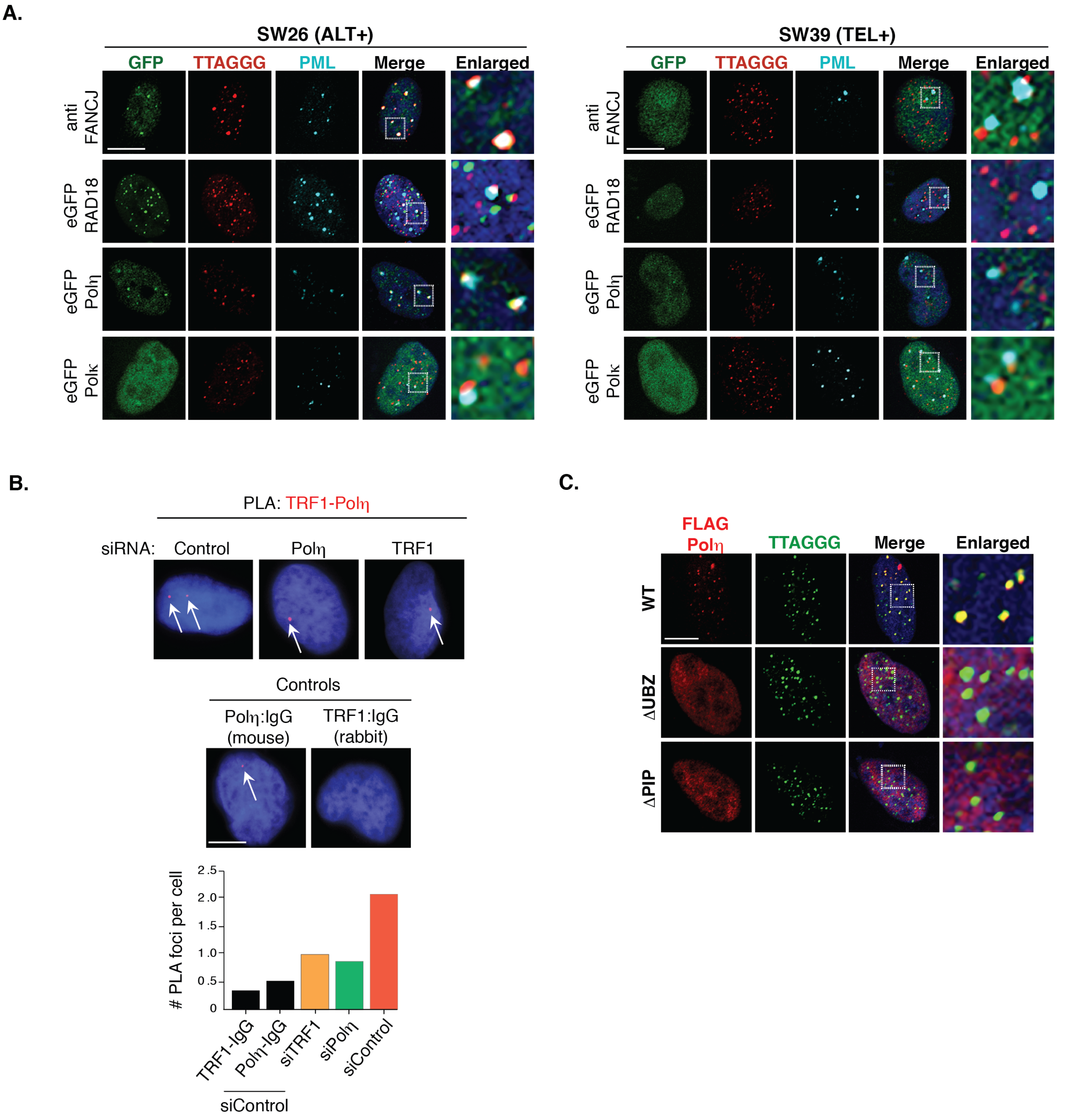

Figure S2. Related to Figure 2. Polη localizes to ALT telomeres. (A) Representative images of the endogenous FANCJ or GFP fusion proteins Polη, RAD18, and Polκ in ALT+ SW26 and TEL+ SW39 together with telomeres (TTAGGG) and PML. Enlarged areas showing co-distributions are shown. Scale bar, 10μM. **(B)** Representative images of TRF1-Polη PLA assay in U2OS cells transfected with control, Polη or TRF1 siRNAs for 72hrs. Images for PLA using control IgG antibodies are shown. Quantifications of the number of TRF1-Polη PLA per nucleus in each experimental condition are shown on the right.
(C) Representative images of FLAG fusion proteins Polη WT, Polη-ΔUBZ and Polη-ΔPIP together with telomeres (TTAGGG) in U2OS. Enlarged areas are shown. Scale bar, 10μM.

Figure S3.

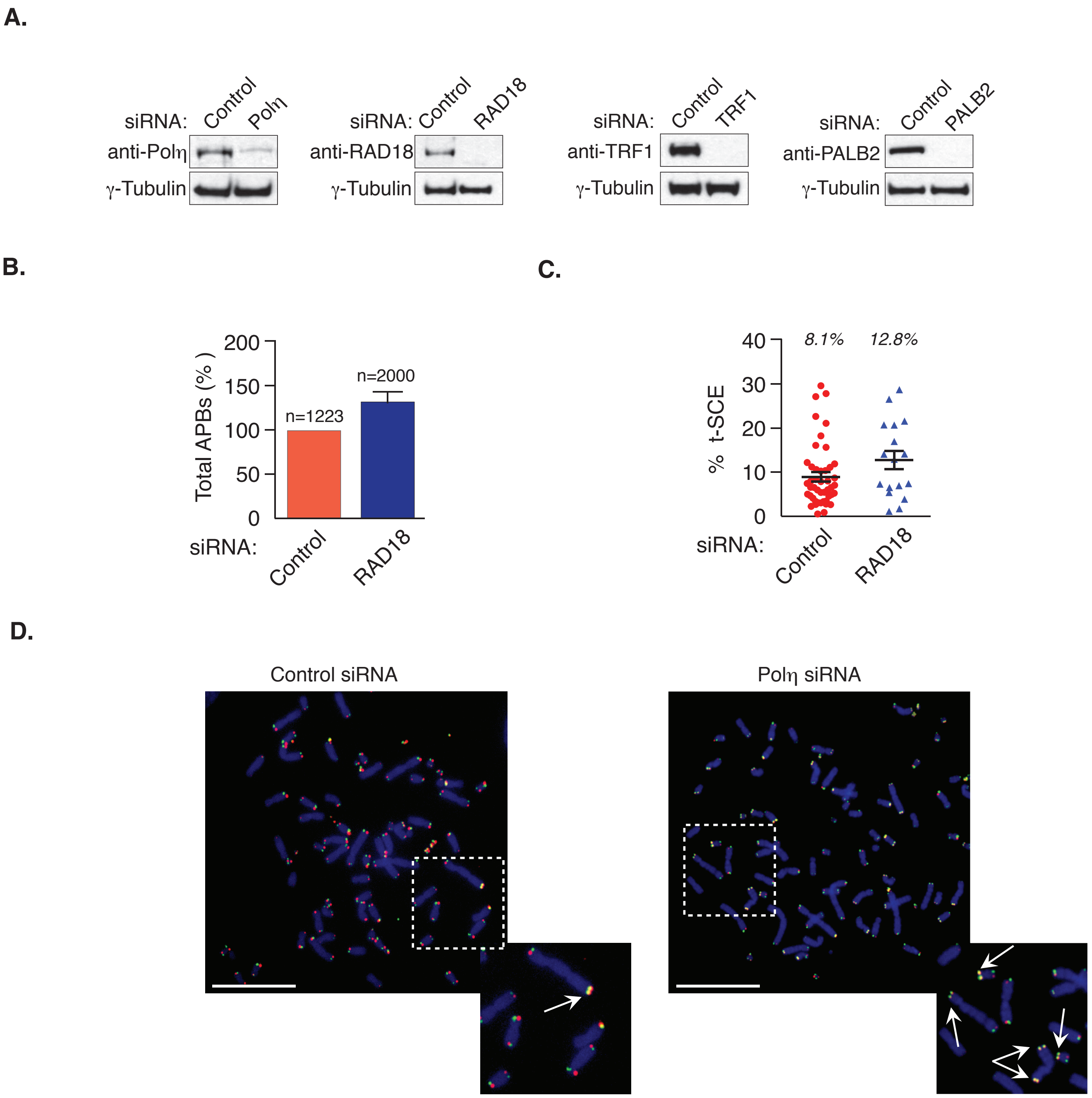

Figure S3. Related to Figures 2 and 3. RAD18 depletion leads to elevated evidence of ALT. (A) Representative western blots of Polη, RAD18, TRF1 and PALB2 in U2OS cells mock-treated or treated with control or siRNAs targeting the indicated protein. γ-Tubulin is used as a loading control. **(B)** U2OS were transfected with RAD18 siRNAs and harvested at 72hrs. IF-FISH was used to analyze the percentage of APBs (co-localization of RPA2, PML and telomeres) as described above. Data represents mean ± SEM of the normalized percentage of cells positive for APB. **(C)** The percent (%) of t-SCEs in control and RAD18 depleted U2OS cells was determined using the COFISH assay, as in Figure 3. **(D)** Representative images from COFISH assays of control and Polη siRNA transfected U2OS cells. Telomeres are shown with CCCTAA (Green) and TTAGGG (Red) PNA probes with metaphase chromosomes shown in blue (DAPI). Enlarged sections beneath are indicated by the dotted boxes. Scale bar, 5μM

Figure S4.

A.

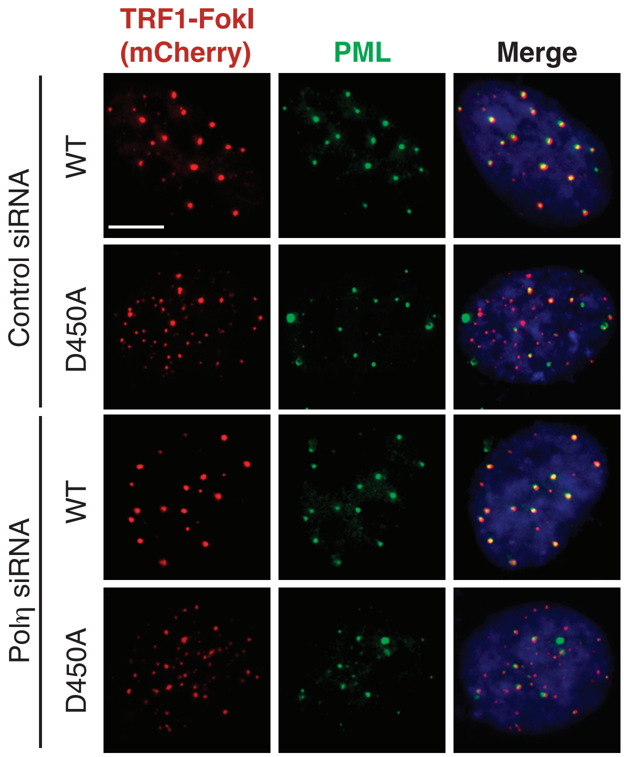

B.

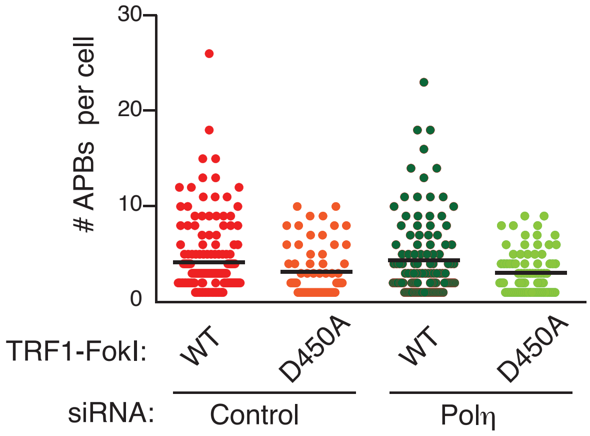

Figure S4. Related to Figure 3. Polη is not recruited to Fok1 nuclease induced telomeric DSBs. (A) Representative images of mCherry-TRF1-FokI WT or FokI mutant D450A and PML in U2OS cells either control or Polη siRNA. **(B)** The number of APBs (co-localization of PML and mCherry-TRF1-FokI) per cell is shown in the graph beneath. Mean is shown as a black bar. Scale bar, 10μM.

Figure S5.

A.

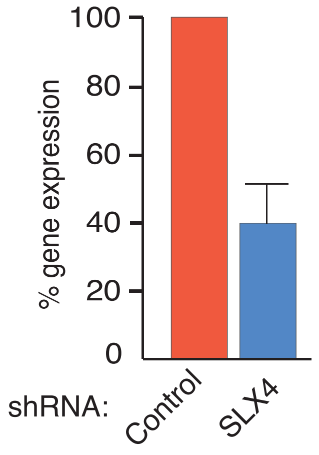

B.

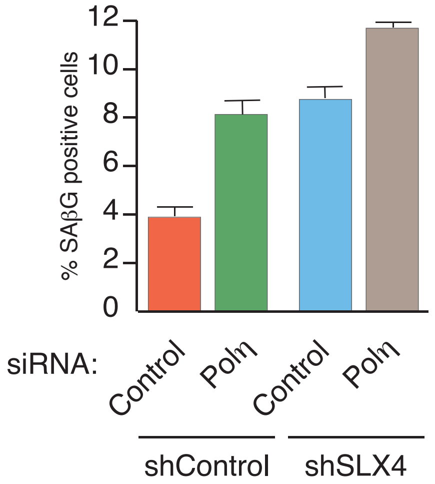

Figure S5. Related to Figure 3 G-H. Validation of SLX4 knockdown. (A) qPCR of SLX4 gene expression in U2OS cells stable expressing a control, non-targeting shRNA and a shRNA against SLX4 mRNA. Abundance of SLX4 mRNA was normalized to the levels of 50S ribosomal subunit, RPLO. **(B)** U2OS shControl and shSLX4 cells transfected with control and Polη siRNA were scored for percent senescence associated beta galactosidase (SAβG) positivity 72hrs following transfection.

Table S1. Related to Figure 1.

List of proteins detected by myc-BiA-TRF1 BioID in U2OS and HeLa LT cells.

Listed proteins shown were identified from triplicate experiments. Average spectral counts (SpC) and standard deviations correspondong to the indicated protein that was detected in triplicate mass specs where myc-TRF1 was used as the bait.

EXPERIMENTAL PROCEDURES

Cell lines and reagents

U2OS, Saos2, VA13, SW26, SW39 and HeLa LT were obtained from ATCC and cultured in Glutamax-DMEM (Life Technologies) supplemented with 10% bovine growth serum. Cells were cultured at low oxygen conditions of 3% O_2 and 7.5% CO_2. Aphidicolin (Sigma-Aldrich) was used at 0.2-0.3μM in culture medium for 12-24hrs as indicated. Hydroxyurea (Sigma-Aldrich) was used at 0.1-1mM in culture medium for the indicated durations.

Antibodies and Plasmids. The following antibodies were obtained as indicated: Anti-TRF2 and anti-TRF1 were obtained from Dr. Jan Karlseder (Salk Institute, CA), Anti-RPA2: mouse, Abcam (ab2175); Anti-PML: rabbit and mouse, Santa Cruz (sc-5621, sc-966); Anti-FANCJ rabbit, Sigma-Aldrich (B1310); Anti-RAD18: rabbit, Bethyl (A301-340A), Anti-RAP1: rabbit, Bethyl (A300-306), Anti-RPA2 (9H8): mouse, Abcam (ab2175); Anti-phospho(S4/8)-RPA2: rabbit, Bethyl (A300-245); Anti-myc (9E11): mouse, Santa Cruz (sc-40); Anti-myc (9E10): mouse, Abcam (ab32); Anti-Polη: rabbit, Abcam (ab17725, ab180703); Anti-PALB2 was a gift from Bing Xia (Rutgers University, NJ), (Anti-γ-Tubulin: mouse, Sigma-Aldrich (T-6557); Streptavidin-HRP conjugated: Thermo Scientific (21126); Streptavidin Alexa Fluor 488 conjugated: Life Technologies (S11223); Anti-FLAG (M2): mouse, Sigma-Aldrich (F1804).

To generate a myc-BirA-TRF1 fusion protein, TRF1 was excised from pLPC-TRF1 using BamHI-EcoRI restriction sites and cloned using the same sites in pLPC-myc-BirA plasmid. All other plasmids were obtained as follows: pLPC-myc-BirA plasmid was a gift from Dr. Eros Lazzerini (Scripps La Jolla, CA). pEGFP-NLS- Polη, pcDNA3.1-FLAG- Polη WT, ΔUBZ and ΔPIP were gifts from Dr. Alan Lehmann (University of Sussex, UK) and described in (Bienko et al., 2010). pEGFP-Polκ was a gift from T Huang (NYU) and described in (Jones et al., 2012). pEGFP-RAD18 was a gift from Dr. Cyrus Vaziri (University of North Carolina, NC) and described in (Durando et al., 2013). pLPC-myc and pLPC-FLAG-TRF1 were a gift from Dr. Jan Karlseder (Salk Institute, CA). WT and nuclease dead (D450A) TRF1-FokI plasmids were a gift from Roger Greenberg (UPenn, Philadelphia).

Proximity-dependent biotinylation and streptavidin capture of biotinylated proteins. U2OS and HeLa cell stably expressing myc-BirA alone or myc-BirA-TRF1 were generated by retroviral infection with particles generated from amphotrophic 293T viral packaging cell lines. Infected cells were selected using puromycin (2mg/ml) for 5-7 days and stable protein expression was validated by western blot and immunofluorescence. Proximity-dependent biotinylation and streptavidin capture of biotinylated proteins was performed as described in (Roux et al., 2012) with minor modifications. Briefly, cells (6 x 10^7) were incubated for 24hrs in complete media supplemented with 50μM biotin (Sigma-Aldrich). Cells were harvest and washed in PBS prior to lysis in 0.2% NP40 buffer to remove cytoplasmic proteins. Cell pellet was lysed in buffer SDS and sonicated to extract nuclear proteins, and finally heated at 98°C for 5mins to denature proteins. Protein samples were incubated with magnetic streptavidin beads (Millipore) overnight at 4°C on a rotary shaker. Next day, beads were washed and finally eluted in 50mM Tris (pH 7.4), 50mM NaCl and 2% SDS together with boiling at 98°C for 5mins.

Mass Spectrometry and Bio-ID of TRF1 associated proteins. Immuno-precipitated samples were separated ~1.5cm on a 10% Bis-Tris Novex mini-gel (Invitrogen) using the MES buffer system. The gel was stained with coomassie and each lane was excised into ten equally sized segments. Gel pieces were processed using a robot (ProGest, DigiLab) as follow: First washes were with 25mM ammonium bicarbonate followed by acetonitrile. Then, reduced with 10mM dithiothreitol at 60°C followed by alkylation with 50mM iodoacetamide at RT. Samples were digested with trypsin (Promega) at 37°C for 4hrs, and then quenched with formic acid. Samples supernatants were analyzed directly without further processing using a nano LC/MS/MS with a Waters NanoAcquity HPLC system interfaced to a ThermoFisher Q Exactive. Peptides were loaded on a trapping column and eluted over a 75mm analytical column at 350nL/min; both columns were packed with Jupiter Proteo resin (Phenomenex). The mass spectrometer was operated in data-dependent mode, with MS and MS/MS performed in the Orbitrap at 70,000 FWHM resolution and 17,500 FWHM resolution, respectively. The fifteen most abundant ions were selected for MS/MS. Data were searched using a local copy of Mascot with the

following parameters: Enzyme: Trypsin; Database: Swissprot Human (forward and reverse appended with common contaminants); Fixed modification: Carbamidomethyl (C); Variable modifications: Oxidation (M), Acetyl (Protein N-term), De-amidation (NQ), Biotin (K); Mass values: Mono-isotopic; Peptide Mass Tolerance: 10 ppm; Fragment Mass Tolerance: 0.02 Da; Max Missed Cleavages: 2. Mascot DAT files were parsed into the Scaffold software for validation, filtering and to create a non-redundant list per sample. Data were filtered 1% protein and peptide level false discovery rate (FDR) and requiring at least two unique peptides per protein. Proteins shared between myc-BirA control and myc-BirA-TRF1 were automatically omitted from consideration.

Co-Immunoprecipitations. U2OS cells stably expressing myc or myc-TRF1 were infected with adenovirus expressing GFP-Polη (Generous gift from Cyrus Vaziri, UNC, described in (Durando et al., 2013)) (MOI 10) for 24hrs. Then, myc-tagged proteins were immunoprecipitated using μMACS c-myc epitope tag Isolation Kit (Miltenyi Biotech) according to manufacturer's instructions. To investigate DNA dependency lysates were processed in the presence or absence of Ethidium Bromide (20mg/ml).

Western blotting. Cells were harvested with trypsin, quickly washed in PBS, counted with Cellometer Auto T4 (Nexcelom Bioscience) and directly lysed in 4X NuPage LDS sample buffer at 10^4 cells per ml. Proteins were gently homogenized using a Nuclease (ThermoFisher), denatured for 10mins at 68°C and resolved by SDS-Page electrophoresis, transferred to nitrocellulose membranes, blocked in 5% milk or BSA and 0.1% Tween for 30mins and probed. For secondary antibodies, HRP-linked anti-rabbit or mouse (Amersham) was used, and the HPR signal was visualized with SuperSignal ECL substrate (Pierce) as per the manufacturer's instructions.

siRNA transfections. For siRNA knockdown we used the On-Target Plus (OTP) siRNA smartpools from Dharmacon (GE). We also used a single siRNA targeting the 3'UTR of Polη mRNA. The sequence of this siRNA oligo is; 5′-GCAATGAGGGCCTTGAACA-3 and was synthesized and purchased from Dharmacon (GE). Briefly, ~200,000 and ~700,000 cells were seeded per well of a 6-well plate and 10cm dish containing growth

medium without antibiotics, respectively. ~2hrs later cells were transfected. siRNAs and Dharmafect were diluted in OptiMEM (Life Technologies). A working siRNA concentration of 50nM was used. We used 2.5ml and 5ml Dharmafect #1 per well and 10cm plate, respectively. Transfection medium was replaced with complete culture media 24hrs later or cells were split for desired application and harvested at 72hrs post transfection, unless otherwise indicated.

Direct Immunofluorescence. All immunofluorescence was performed as described in (O'Sullivan et al., 2014). Cells on glass coverslips were washed twice in PBS and fixed with 2% PFA for 10mins. Cells were permeabilized with 0.1%(w/v) sodium citrate and 0.1% (v/v) Triton X-100 for 5mins and incubated with fresh blocking solution (1mg/ml BSA, 10% normal goat serum, 0.1% Tween) for at least 30mins. Primary antibodies were diluted in blocking solution and added to cells for 1hr at RT or overnight at 4°C. Next, cells were washed in 3 times with PBS for 5mins and incubated with Alexa coupled secondary antibodies (568nm, 647nm) (Life Technologies) for 1hr at RT. Then, cells were washed 3 times with PBS and mounted on slides with Prolong Gold Anti-fade reagent with DAPI (Life Technologies). Once the Prolong Anti-fade has polymerized and cured for ~24hrs cells were visualized by conventional florescence with 20X and/or 63X Plan λ objective (1.4 oil) using a Nikon 90i or Nikon A1R Spectral confocal microscope.

IF-FISH. IF-FISH was performed as described in (O'Sullivan et al., 2014). Briefly, after secondary antibody incubation, cells were washed as above but then the IF staining was fixed with 2% PFA for 10mins. PFA was washed off with PBS and coverslips dehydrated with successive washes in 70%, 95% and 100% EtOH for 3mins, allowed to air dry completely. Next, the coverslips were mounted on glass slides with 15ml per coverslip of hybridization mix (70% deionized Formamide, 1 mg/ml of Blocking Reagent [Roche], 10mM Tris-HCl pH 7.4) containing Alexa 488-$(CCCTAA)_n$ PNA probe. DNA was denatured by setting the slides on a heating block set to 72°C for 10mins and then incubating for at least 4hrs or overnight at RT in the dark. The coverslips were then washed twice for 15mins with Wash Solution A (70% deionized formamide and 10 mM Tris-HCl pH 7.2) and three time with Solution B (0.1M Tris-HCl pH 7.2, 0.15M NaCl

and 0.08% Tween) for 5mins at RT. EtOH dehydration was repeated as above and finally the samples were mounted and analyzed as mentioned above.

Chromosome Orientation FISH. CO-FISH was performed as described in (O'Sullivan et al., 2014), with the variation that cells were incubated with BrdU and BrdC simultaneously for ~12hrs, and hybridization was performed with Alexa 488-$(CCCTAA)_n$ and Alexa 568-$(TTAGGG)_n$ PNA probes. In brief, cell cultures were incubated with 7.5mM BrdU and 2.5mM BrdC for ~12hrs. After removal of nucleotide analogs, colcemid (Gibco) was added for ~2hrs, cells were harvested by trypsinization, swelled in 75mM KCl and fixed in 70% Methanol: 30% Acetic Acid. Samples are stored at -20°C for days. Metaphase chromosomes were spread by dropping onto washed slides, then RNase A (0.5 mg/ml) and pepsin treated. Slides were incubated in 2X SSC containing 0.5mg/ml Hoechst 33258 for 15mins in the dark and irradiated for 40mins (5.4 x 10^5 J/m2, energy 5400) at in a UV Stratalinker 2400 (Stratagene). The nicked BrdU/C substituted DNA strands are degraded by Exonuclease III digestion. The slides were then washed in PBS, dehydrated by EtOH washes and allowed to air dry completely. The remaining strands were hybridized with fluorescence labeled DNA probes of different colors, specific either for the positive telomere strand $(TTAGGG)_n$ (polymerized by lagging strand synthesis) (Alexa-488, green color), or the negative telomere strand $(CCCTAA)_n$, (polymerized by leading strand synthesis) (Alexa-568, red color). Prior to hybridization of the first PNA, DNA is denatured by heating at 72°C for 10mins, as in IF-FISH, and then incubated for 2hrs at RT. Slides were washed for 15mins with Wash Solution A (see IF-FISH), dried and then incubated with the second PNA for 2hrs at RT. The slides were then washed again twice for 15mins with Wash Solution A and 3 times with Wash Solution B (see IF-FISH) for 5mins at RT. The second wash contained DAPI (0.5μg/ml). Finally, cells were dehydrated in EtOH as above and mounted (Vectashield). The resulting chromosomes show dual staining and allow distinction between leading and lagging strands. Metaphase chromosomes were visualized by conventional florescence microscope with a 63X Plan λ objective (1.4 oil) on a Nikon 90i microscope.

Proximity Ligation Assay (PLA)

U2OS cells were transfected with siRNA as indicated and 24hrs later cells were seed on coverslip with or without 0.3mM aphidicolin or 100mM hydroxyurea for 24hrs. Cells were washed twice with PBS, fixed with 3% formaldehyde, permeabilized with 0.5% Triton X-100 and blocked with blocking buffer (Duolink®-Sigma-Aldrich). Coverslips were incubated with primary antibodies for 2hrs at RT: Polη (Rabbit, Abcam ab17725 1/250), TRF1 (Mouse, Abcam ab10579 1/250), normal mouse IgG1 (Santa Cruz sc-2025) and normal rabbit IgG (Santa Cruz sc-2027). Proximity ligation was performed utilizing the Duolink® In Situ Red Starter Kit Mouse/Rabbit (Sigma-Aldrich) according to the manufacturer's protocol. The oligonucleotides and antibody-nucleic acid conjugates used were those provided in the Sigma-Aldrich PLA kit (DUO92101). Fluorescence was detected using a Nikon eclipse microscope with a 63X Plan λ objective (1.4 oil) and halogen lamp. Images were quantified in triplicate using NIS-Elements Advanced Research software (Nikon) as foci per nucleus, defined as the number of interaction points counted per nucleus.

Analysis of DNA synthesis on metaphase chromosomes.

Telomeres with delayed replication were quantified using an in situ 5-ethynyl-2'-deoxyuridine (EdU) incorporation assay combined with telomeric FISH. U2OS cells were seed on coverslip and siRNA transfected. 24hrs later, cells were treated with 0.3µM aphidicolin for 24hrs. Cells were incubated with 10µM EdU for 40mins and immediately fixed in PBS containing 4% PFA for 20mins and then permeabilized in PBS-0.5% Triton X-100 for 30mins. EdU was detected by click reaction using the Click-iT EdU-Alexa 488 Imaging kit (Life Technologies), according to the manufacturers' instructions. EdU staining was fixed with 4% PFA for 10mins and subsequently FISH was performed as described above with Cy3-$(CCCTAA)_n$ PNA probe. Images were acquired using a LSM 780 (Zeiss) confocal microscope with a 63X Plan-ApoChromat objective (1.4 oil) and argon laser at 488 nm, DPSS laser at 561nm, and helium laser at 633nm.

FokI mediated induction of telomeric DSBs.

U2OS cells were transfected with siRNA as indicated. 48hrs later, the same cells were transfected with mCherry-TRF1-FokI-ER-DD WT or the inactive FokI mutant D450A. FokI transgenes were induced with 4-OHT (2 mM) and Shield1 ligand (0.5mM) as previously described (Cho et al., 2014). Cells were processed for immunofluorescence using PML antibody as described above. APBs (mCherry-TRF1 and PML co-localizations) were quantified in triplicate using NIS-Elements Advanced Research software (Nikon) as #APBs per cell.

C-Circle Assay. The protocol for C-circle amplification was employed as (O'Sullivan et al., 2014). Briefly, genomic DNA was purified, digested with *Alu*I and *Mbo*I and cleaned up by phenol-chloroform extraction and precipitation. DNA was diluted in ultraclean water and concentrations were exhaustively measured to the indicated quantity (generally, 30, 15, 7.5ng) using a Nanodrop (ThermoFisher). Samples (10µl) were combined with 10µl BSA (NEB; 0.2 mg/ml), 0.1% Tween, 0.2mM each dATP, dGTP, dTTP and 1 × Φ29 Buffer (NEB) in the presence or absence of 7.5U ΦDNA polymerase (NEB). Samples were incubated at 30°C for 8hrs and then at 65°C for 20mins. Reaction products were diluted to 100µl with 2 × SSC and dot-blotted onto a 2 × SSC-soaked nylon membrane. DNA was UV cross-linked onto the membrane and hybridized with a P_{32} end-labelled $(CCCTAA)_4$ oligo probe to detect C-circle amplification products. All blots were washed, exposed to PhosphoImager screens, scanned and quantified using a Typhoon 9400 PhosphoImager (Amersham/GE Healthcare). In all reactions, when Φ29 was omitted as a negative control, U2OS DNA was used.

T-Circle Assay. The protocol for T-circle amplification was performed as in (Zellinger et al., 2007). Genomic DNA was prepared as above. Then DNA was denatured at 96°C for 5 mins and annealed to 50pmol of the telomere-specific primer $(CCCTAA)_4$ for 30–60mins at RT in a buffer containing 200mM Tris–HCl pH 7.5, 200mM KCl and 1mM EDTA in a total volume of 20ml. Half (10ml) of the annealed reaction was incubated at 30°C for 12hrs in the presence of dNTPs (0.2mM each), BSA (NEB; 0.2mg/ml), 1×Φ29 buffer (NEB) and 7.5U Φ29 DNA polymerase (NEB) in a total volume of 20ml, and the

remaining half was incubated under identical conditions with one exception; the Φ29 DNA polymerase was excluded from the reaction. Both sets of reactions that did or did not include Φ29 were further incubated at 65 °C for 20mins. The samples were then electrophoresed on 0.5% agarose gels at 120V for 1hr then 38–40V overnight in 0.5×TBE buffer in the presence of ethidium bromide. After electrophoresis the gels were dried at 50°C, denatured and hybridized P_{32} end-labeled $(TTAGGG)_4$ oligo probe. All blots were washed and exposed to PhosphoImager screens.

Telomere chromatin immunoprecipitation (ChIP). ChIP was performed as described in (O'Sullivan et al., 2014).

Flow Cytometry. Cells to be used for flow cytometry were fixed in 70% EtOH and stained directly with propidium iodide (Life Technology) containing RNase A (100ng/ml). Cell cycle profiles were analyzed using BD Accuri C6.

D-loop assay. <u>*Proteins.*</u> Recombinant human DNA polymerase η was purchased from Enzymax (Lexington, KY). Recombinant human Polδ (four subunits) was purified as described previously (Xie et al., 2002). Recombinant PCNA and RFC were purified from *Escherichia coli* overproduction strains as described (Ayyagari et al., 2003). Recombinant RecA protein was purchased from New England Biolabs (Ipswich, MA).

<u>*DNA substrates.*</u> All unmodified oligonucleotides were purchased from Integrated DNA Technologies (Coralville, IA) and were purified by PAGE or HPLC by the manufacturer. Oligonucleotides were 5′ end labelled using [γ-^{32}P] ATP (3000Ci/mmol) (Perkin Elmer, Waltham, MA) with OptiKinase (Affymetrix, Santa Clara, CA) following the manufacturer's instructions. The plasmid used for preparing the 120-bp telomeric and non-telomeric D-loops was as described in (Hile et al., 2000), and was purified by two rounds of ethidium bromide saturated CsCl equilibrium gradient ultracentrifugation (Loftstrand Labs, Gaithersburg, MD). The 120 mer invading strand oligonucleotides used for D-loop construction are 5′ AATTCTCATTTTACTTACCTGACGCTATTAGCAGTGCAGGCTGCGCGTTTAGG

GTTAGGGTTAGGGTTAGGGTTAGGGTTAGGGTTAGGGTTAGGGTTAGGGTTA GGGTCTCGAGGCCAT and 5′AATTCTCATTTTACTTACCTGACGCTATTAG CAGTGCTCTTCGGCCAGCGCCTTGTAGAAGCGCGTATGGCTTCGTACCCCGGC CATCCACACGCGTCTGCGTTCGACCAGGCTGCGCGT. The plasmid based D-loop substrates were constructed as described previously (Bachrati and Hickson, 2006). Briefly, 5′ end radiolabeled invading strand oligonucleotide (5.0 pmol) was incubated in RecA buffer (500mM Tris, pH 7.5, 150mM $MgCl_2$, 10mM DTT, 1 mg/mL BSA) with RecA enzyme (0.2 µg/µL) in the presence of ATP (3.0 mM), Phosphocreatine (28.6mM) and Creatine Phosphokinase (14.3U/mL) (Sigma Aldrich, St. Louis, MO) for 5mins at 37°C. 10 µg of the supercoiled plasmid was then added and incubated for another 3mins at 37°C. The reaction was terminated using stop buffer containing 10 mg/mL Proteinase K (New England Biolabs, Ipswich, MA) and 3.0% SDS (Thermo Fisher, Waltham MA) followed by incubation for 30mins at 37°C. The D-loop constructs were PAGE purified using 4.5% (37.5:1) polyacrylamide gels at 200V for 2hrs. The D-loop substrates were visualized by phosphorimager analysis, excised, and subjected to electro-elution using dialysis D-tubes (Novagen, Madison, WI) in 1xTBE for 3 hrs at 4°C and 120V. Samples were then concentrated using micron-30 columns (Amicon) and exchanged into dialysis buffer (10 mM Tris, pH 7.5, 10 mM $MgCl_2$). Product purity and yield were determined by analysis on 4–20% native polyacrylamide gel, followed by quantification using Typhoon phosphorimager and ImageQuant software (GE Healthcare, Piscataway, NJ).

D-loop extension assay. Reactions (10µl) for Polη alone contained 20fmol of D-loop in 25mM potassium phosphate buffer pH7.2, 5mM $MgCl_2$, 250µM dNTPs, 5mM DTT and 100µg/mL BSA. Reactions were initiated by adding 20–320fmol of Polη as indicated in the figure legends, and were incubated at 37°C for 1hr followed by termination using an equal volume of 95% formamide and 5mM EDTA. The dual polymerase reactions (10µl) contained 20fmol of D-loop substrate in 40mM Tris, pH 7.8, 10mM $MgCl_2$, 1mMDTT, 200µg/mL BSA, 5mMNaCl, 0.5mM ATP and 250µM dNTP. Where indicated the substrate was pre-incubated with 500fmol of PCNA and 40fmol of RFC for 5mins at 37°C. The reactions were initiated by adding either 20fmol of Polδ or 40fmol of Polη and incubated for 15, 45, or 60mins. After 15mins the reactions were supplemented with

either 40fmol Polη or 20fmol Polδ, respectively, and continued for another 45mins as indicated in the figure legends. Following termination, the reactions were boiled at 100°C for 10mins and snap cooled in ice before loading on a 10% denaturing polyacrylamide gel for 3hrs under constant current of 38W. The gel was exposed overnight and visualized using Typhoon phosphorimager. Unused primer was quantified using ImageQuant software and background corrected using the no enzyme control reactions. To analyze the extent of DNA synthesis, the amount of radioactivity in bands for products was divided by the total amount of radioactivity in the lane.

REFERENCES.

Ayyagari, R., Gomes, X.V., Gordenin, D.A., and Burgers, P.M.J. (2003). Okazaki fragment maturation in yeast. I. Distribution of functions between FEN1 AND DNA2. J. Biol. Chem. *278*, 1618–1625.

Bachrati, C.Z., and Hickson, I.D. (2006). Analysis of the DNA unwinding activity of RecQ family helicases. Meth. Enzymol. *409*, 86–100.

Bienko, M., Green, C.M., Sabbioneda, S., Crosetto, N., Matic, I., Hibbert, R.G., Begovic, T., Niimi, A., Mann, M., Lehmann, A.R., et al. (2010). Regulation of translesion synthesis DNA polymerase eta by monoubiquitination. Mol. Cell *37*, 396–407.

Cho, N.W., Dilley, R.L., Lampson, M.A., and Greenberg, R.A. (2014). Interchromosomal homology searches drive directional ALT telomere movement and synapsis. Cell *159*, 108–121.

Durando, M., Tateishi, S., and Vaziri, C. (2013). A non-catalytic role of DNA polymerase η in recruiting Rad18 and promoting PCNA monoubiquitination at stalled replication forks. Nucleic Acids Res. *41*, 3079–3093.

Hile, S.E., Yan, G., and Eckert, K.A. (2000). Somatic mutation rates and specificities at TC/AG and GT/CA microsatellite sequences in nontumorigenic human lymphoblastoid cells. Cancer Res. *60*, 1698–1703.

Jones, M.J., Colnaghi, L., and Huang, T.T. (2012). Dysregulation of DNA polymerase κ

recruitment to replication forks results in genomic instability. Embo J. *31*, 908–918.

O'Sullivan, R.J., Arnoult, N., Lackner, D.H., Oganesian, L., Haggblom, C., Corpet, A., Almouzni, G., and Karlseder, J. (2014). Rapid induction of alternative lengthening of telomeres by depletion of the histone chaperone ASF1. Nat. Struct. Mol. Biol. *21*, 167–174.

Roux, K.J., Kim, D.I., Raida, M., and Burke, B. (2012). A promiscuous biotin ligase fusion protein identifies proximal and interacting proteins in mammalian cells. J. Cell Biol. *196*, 801–810.

Xie, B., Mazloum, N., Liu, L., Rahmeh, A., Li, H., and Lee, M.Y.W.T. (2002). Reconstitution and characterization of the human DNA polymerase delta four-subunit holoenzyme. Biochemistry *41*, 13133–13142.

Zellinger, B., Akimcheva, S., Puizina, J., Schirato, M., and Riha, K. (2007). Ku suppresses formation of telomeric circles and alternative telomere lengthening in Arabidopsis. Mol. Cell *27*, 163–169.
